# Molecular characterization of the grape seeds extract’s effect against chemically induced liver cancer: *In vivo* and *in vitro* analyses

**DOI:** 10.1038/s41598-018-19492-x

**Published:** 2018-01-19

**Authors:** Alaaeldin Ahmed Hamza, Gehan Hussein Heeba, Hanan Mohamed Elwy, Chandraprabha Murali, Raafat El-Awady, Amr Amin

**Affiliations:** 1 Hormone Evaluation Department, National Organization for Drug Control and Research, Giza, Egypt; 2 Department of Pharmacology and Toxicology, Faculty of Pharmacy, Minia University, El-Minia, Egypt; 3 Analytical Chemistry Department, NODCAR, Giza, Egypt; 4 Biology Department, UAE University, Al-Ain, UAE; 5 Department of Pharmacy Practice & Pharmacotherapeutics and Sharjah Institute for Medical Research, University of Sharjah, Sharjah, UAE; 6 Zoology Department, Cairo University, Giza, Egypt

## Abstract

The purpose of this study was to investigate the anti-cancer property of grape seed extract (GSE) during early stages of developing liver cancer using a two-stage carcinogenic model combining diethylnitrosamine (DEN) and 2-Acetyl Aminofluorene (2-AAF). Administration of GSE at doses 25, 50 and 100 mg/kg per day started at the beginning of promotion periods and continued for 14 weeks. GSE dramatically inhibited pre-neoplastic foci formation as well as significantly decreased the number and the area of placental glutathione-S-transferase in livers of DEN-2AAF-treated rats by approximately 4 & 10 fold deductions, respectively. GSE’s effects were associated with induced apoptosis, reduced cell proliferation, decreased oxidative stress and down regulation of histone deacetylase activity and inflammation makers, such as cyclooxygenase 2, inducible nitric oxide synthase, nuclear factor-kappa B-p65 and p- phosphorylated tumor necrosis factor receptor expressions in liver. GSE treatment also decreased the viability of HepG2 cells and induced early and late apoptosis through activating caspase-3 and Bax. Furthermore, GSE induced G2/M and G1/S cell cycle arrest. The present study provides evidence that the GSE’s anticancer effect is mediated through the inhibition of cell proliferation, induction of apoptosis, modulating oxidative damage and suppressing inflammatory response.

## Introduction

Hepatocellular carcinoma (HCC) remains a leading cause of cancer-related death both in developed and under-developed countries^1^. Chronic infection with hepatitis B and C are the main causes of HCC^2^. Other factors that contribute to the formation of HCC include fatty liver disease, iron overload, alcoholism and exposure to environmental carcinogens^3^. One of the most common carcinogens is diethylnitrosamine (DEN), which is widely used in the surrounding of everyday life, in tobacco, smoke, processed food, gasoline, and cosmetics^4^.

Chemoprevention of cancer especially by natural compounds is a promising strategy to protect against various stages of cancer development^5–7^. Total plant extracts have been of a particular interest mainly because of the synergistic effects of the cocktail of plant metabolites and their multiple points of intervention during chemoprevention^7,8^. The development of pre-neoplastic foci of altered hepatocytes (FAH) was exploited as short-term bioassays to assess the chemopreventive potential of natural products against cancer formation^9^. Thus, inhibiting or suppressing the development of pre-neoplastic FAH by natural products may lead to diminishing the subsequent progression to liver cancer. One particular plant product that has gained much attention is grape seed extract (GSE). Grapes (*Vitis vinifera*) are rich in polyphenols, with 60–70% of grape polyphenols being found in the seeds, which are available as a nutraceutical agent. The consumer’s interest in GSE has been primarily due to its high content of antioxidants in the form of flavonoids, polyphenols and proanthocyanidins^10,11^. GSE has been shown to possess potent cardioprotective, hepatoprotective, antidiabetic, anti-mutagenic and anti-inflammatory properties^10–12^. Moreover, GSE has shown promising chemopreventive and anticancer effects in various cancer cells and in a wide variety of animal tumor models such as skin, colorectal, prostate and breast cancers^13–15^. So far, limited efforts have been set forward to investigate, mainly *in vivo*, the effect of GSE against liver cancer^16,17^.

Cancer development is a multifactorial and multistage process consisting of three distinct phases: initiation, promotion and progression^8^. In experimental carcinogenesis, pre-neoplastic FAH is considered the earliest indicative morphological changes prior to the appearance of HCC. Similar progression has been described in human hepatocarcinogenesis^9^. The initiation–promotion model of cancer development used in this study mimics the early events of the latent period of human carcinogenesis. The initiation stage of cancer development can be produced by the administration of single dose of DEN, a carcinogen that causes DNA ethylation and mutagenesis^9^. DEN has been used to induce lesions in rats that mimic different types of benign and malignant tumors in human^18^. If rats are older than 2 weeks, a tumor promoter is required following DEN administration to enhance hepatocarcinogenesis. In the two-stage model protocol, initiation and promotion steps are important in developing HCC where promotor induce clonal expansion of initiated cells^19^. The single dose administration of DEN exposes the animals to a reduced external effect and mimics a pathophysiological progression of HCC. Extended protocols that involves repeated DEN administration for longer time induce tumour formation at higher rate; however, those models are often influenced by the multiple DEN injections^20^. Accordingly, well–designed mechanism-based preclinical studies in animal models are needed to establish the dietary GSE as an effective chemopreventive agent against HCC. In the present model, initiation is followed by a growth stimulus (Fasting and re-feeding) during treatment with promoting agent such as 2-Acetyl Aminofluorene (2-AAF) that induces selective proliferation of the initiated cell population over non initiated cells in the target tissue^9,21^.

This well-described model of HCC was utilized to study the mechanism of the anti-cancer action of GSE during the early stages of hepatocellular tumor promotion by evaluating its antioxidant, pro-apoptotic, anti-proliferative, histone deacetylase (HDAC) inhibitory and anti-inflammatory effects. In addition, HepG2 cells were used here to shed more light on the mechanism by which GSE exerts its anticancer activity against liver cancer *in vitro*.

## Results

### Phytochemical composition and *in vitro* antioxidant properties

The *in vitro* antioxidant capacity of GSE was evaluated with ferric reducing antioxidant power (FRAP),2-azino-bis(3-ethylbenzothiazoline-6-sulfonate) (ABTS), 1,1-diphenyl-2-picrylhydrazyl (DPPH) and β-carotene assays (Table 1). The ascorbic acid equivalent antioxidant capacities of the GSE were 0.68, 1.06 and 1.10 mmol/g in FRAP, ABTS and DPPH assays, respectively. Furthermore, the free radical scavenging activity as measured by the ABTS and DPPH assays, were (IC_50_ = 15.76 µg /mL) and (IC_50_ = 29.25 µg /mL), respectively. This antioxidant capacity was higher than the vitamin E (Trolox).

**Table 1:** *In vitro* antioxidant properties of GSE.

Assay	FRAP Assay	ABTS Assay		DPPH Assay		B-Carotene Assay
	TAC (mmol/g)	TAC (mmol/g)	IC_50_ (µg /mL)	TAC (mmol/g)	IC_50_(µg /mL)	IC_50_ (µg /mL)
GSE	0.68 ± 0.02	1.06 ± 0.02	15.76 ± 0.16	1.10 ± 0.04	29.25 ± 2.25	168.30 ± 1.70
Trolox	0.40 ± 0.01	0.28 ± 0.01	64.42 ± 1.44	0.41 ± 0.02	46.03 ± 0.98	183.11 ± 3.11

Total antioxidant activity of GSE is expressed as ascorbic acid equivalents (mmol/g of dry extract). IC_50_ is the concentration of extract that is able to scavenge free radicals by 50%. Values are means ± s.e.m. of three experiments.

GSE also possessed high amount of total phenolic (141.26 mg gallic acid/g) and flavonid (68.16 mg quercetin/g) contents (Table 2). High-performance liquid chromatography (HPLC) analysis revealed that the Catechin (33.44) and epicatechin (13.03) were the most important phenolic compounds of GSE. Data from the quantitative analysis of GSE using HPLC-Coupled with photodiode array detection, are presented in Table 2. The components of the catechin, and epicatechin were identified by comparison with the retention times of standards analyzed under identical analytical conditions, while the quantitative data were calculated from their respective calibration curves.

**Table 2:** Polyphenolic contents of GSE.

Assay	Total polyphenols	Total flavonoids	Catechin	Epicatechin
	(mg gallic acid/g)	(mg quercetin/g)	(mg /g)	(mg/g)
GSE	141.26 ± 0.50	68.16 ± 0.23	33.43 ± 0.05	13.03 ± 0.01

Values are means ± s.e.m. of three experiments.

### *In vivo* hepatic antioxidant status of GSE

**Figure 1:**
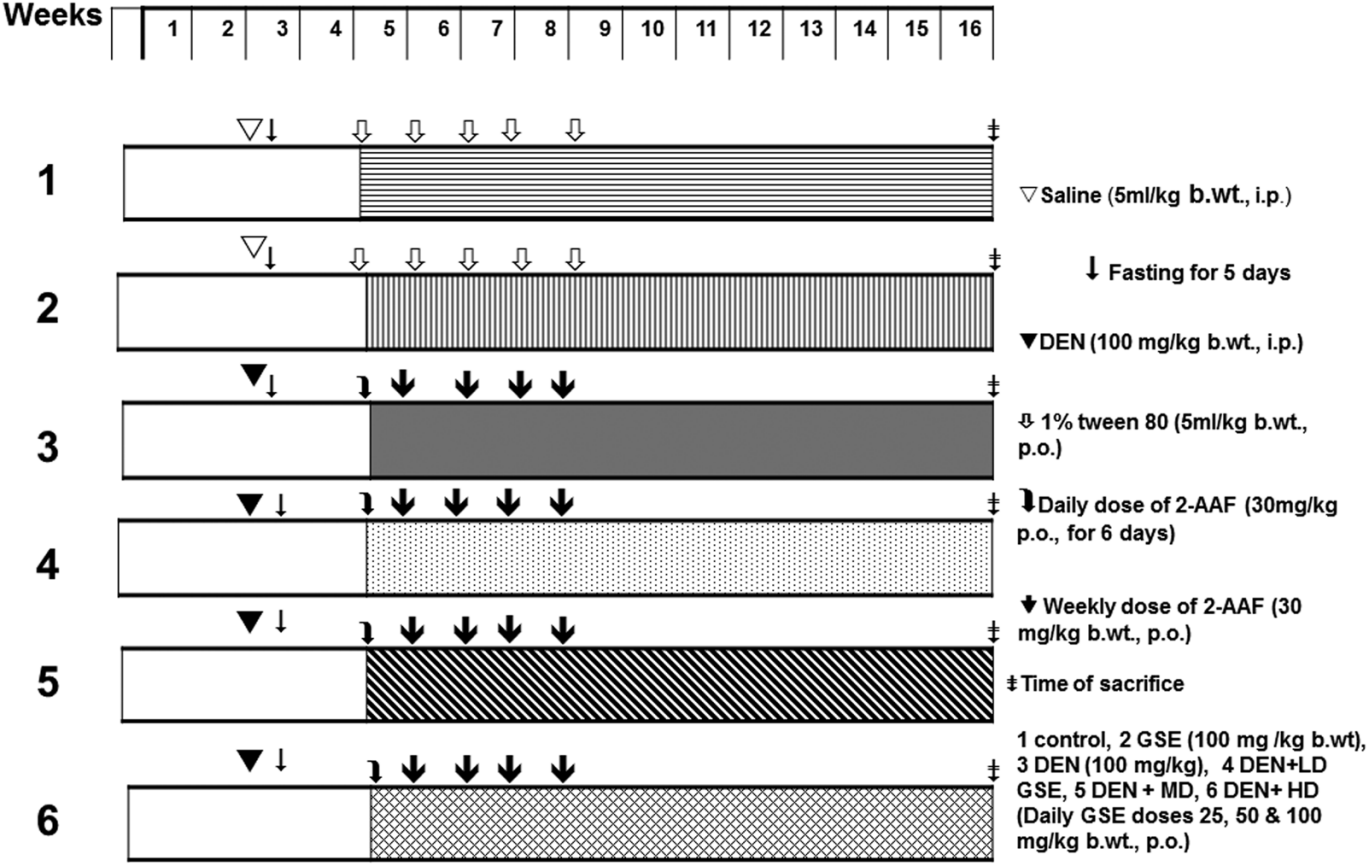
Schematic diagram showing the experimental design to induce and treat liver cancer *in vivo*. (1) Control, (2) GSE alone, (3) DEN-2AAF alone, (4) DEN-2AAF + GSE-LD, (5) DEN-2AAF + GSE-MD, (6) DEN-2AAF + GSE-HD.

An HCC animal model was developed to investigate the anti-HCC effect of GSE (Fig. 1). The antioxidant activity of GSE was also evaluated *in vivo* (Table 3), which shows the antioxidant status of GSE on liver tissues of both control and experimental animal groups. Group 2 demonstrated no significant changes compared to group 1 in terms of the activity of myeloperoxidase (MDA), P.carbonyl, catalase (CAT), and superoxide dismutase (SOD). Nevertheless, group 3 demonstrated a notable change in all oxidative stress markers. When compared to group 1, the levels of MDA, and P.carbonyl as well as the activity of CAT were significantly elevated, and the activity of SOD had decreased. Such changes can be explained by DEN-2AFF-induced hepatic oxidative stress and damage. Pretreatment with GSE significantly attenuated the level changes of these oxidative stress markers. Both medium and high doses of GSE abolished DEN-2-AAF-induced oxidative stress more effectively than the lower dose.

**Table 3:** *In vivo* antioxidant status of GSE and its inhibitory effect on DEN-2AAF-induced oxidative stress in male rats.

Groups	MDA	P.Carbonyl	CAT	SOD
**Control**	0.62 ± 0.01	1.73 ± 0.04	141.98 ± 0.71	2.99 ± 0.01
**GSE**	0.62 ± 0.02	1.71 ± 0.03	142.89 ± 1.83	3.08 ± 0.04
**DEN-2AAF**	0.78 ± 0.02^a^	2.13 ± 0.14^a^	163.05 ± 4.72^a^	2.63 ± 0.15^b^
**DEN-2AAF + GSE LD**	0.59 ± 0.02	1.85 ± 0.04	142.35 ± 1.73	3.18 ± 0.07
**DEN-2AAF + GSE MD**	0.66 ± 0.01	1.78 ± 0.06	141.73 ± 1.68	3.04 ± 0.04
**DEN-2AAF + GSE HD**	0.66 ± 0.01	1.77 ± 0.06	146.01 ± 2.57	3.03 ± 0.06

Values are presented as mean ± s.e.m. (n = 6). Concentration is expressed as nmol/mg protein for MDA and P.Carbonyl. Activity is expressed as unit/mg protein for CAT and SOD. Significance was determined by one-way analysis of variance followed by Dunnett’s t-test: aP < 0.001, bP < 0.015 vs. control group.

### GSE inhibited DEN-2AAF-induced FAH formation and GST-p expression

**Figure 2:**
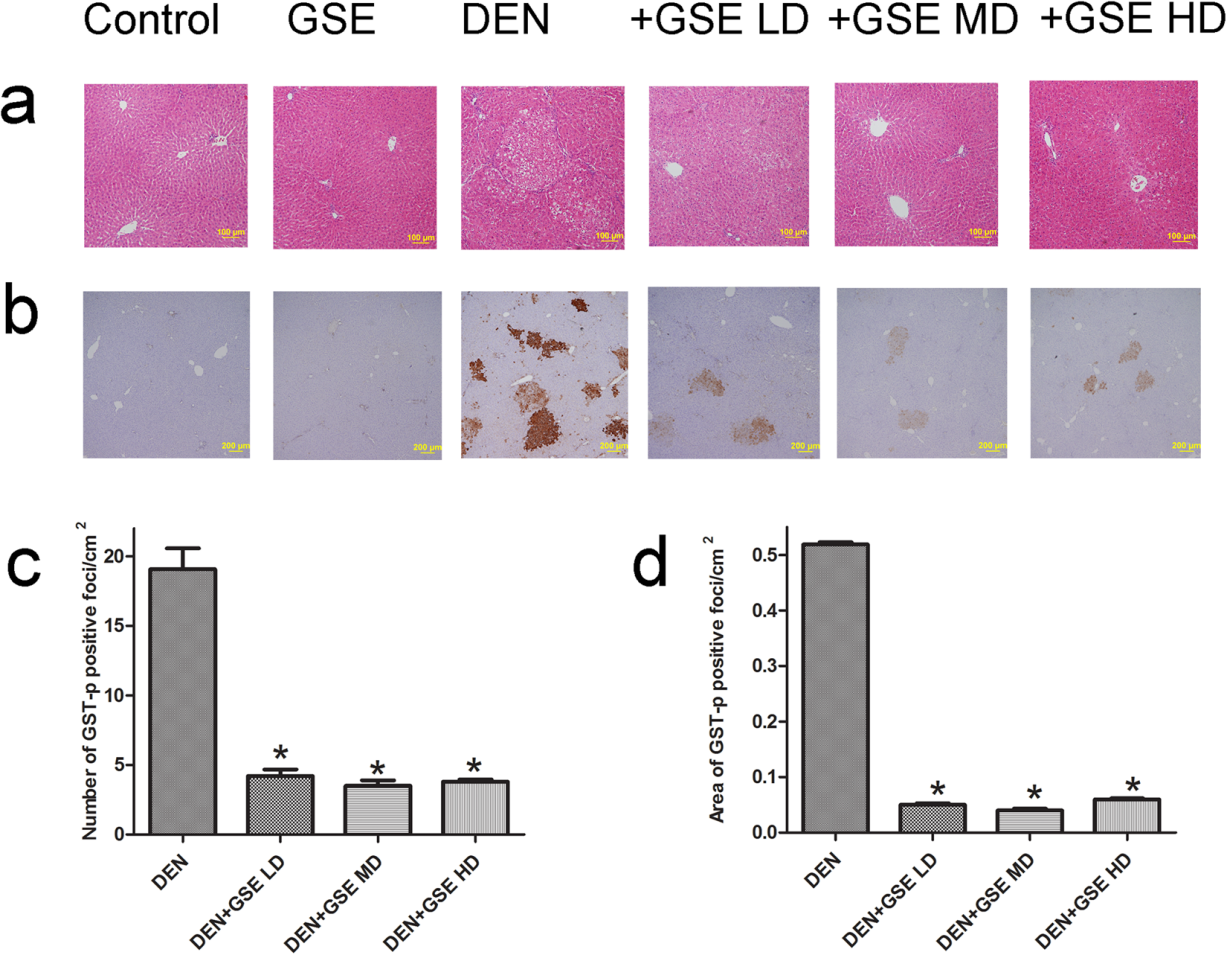
GSE inhibits DEN-2AAF-induced induction of FAH and of GST-p expression in liver. (**a**) Representative Images of hematoxylin and eosin-stained sections in the livers of all groups studied (Scale bar = 100 µm). FAH was observed in liver section from DEN-2AAF-treated group. (**b**) Representative Images of immunohistochemical stained liver sections with GST-p of all groups studied (Scale bar = 200 µm). The lower panel shows quantitative analysis of GST-p-positive foci (**c**) and the quantitative analysis of area of GST-p-positive foci (d). The number and area (mm^2^) of GST-p-positive foci was reduced in GSE-treated rats. Significance was determined by one-way analysis of variance followed by Dunnett’s t-test: *P < 0.05 vs. DEN-2AAF group. Data are represented as mean ± s.e.m.

Histological examination showed pre-neoplastic liver foci in animals treated with DEN-2AAF alone (Fig. 2a and Supplementary Fig. 1). These pre-neoplastic foci are composed of large, irregular and pale hepatocytes with large hyperchromatic nuclei and extensive vacoulations in the cytoplasm which represents the classical FAH structure. GSE treatment in DEN-2AAF-induced rats improved the hepatocellular architecture with more regular hepatocytes and less FAH. These findings showed the dramatic protection offered by GSE against hepatocellular carcinoma. Induction of GST-p is considered as an early biomarker of hepatocarcinogenesis and a well–accepted end-point lesion in liver carcinogenicity^9^. GST-p foci larger than 15 cells were measured using color image processor. The number and areas of foci /cm^2^ of liver sections were calculated. In animals treated with DEN-2AAF, the number of GST-p positive foci and the area per cm^2^ were dramatically increased (Fig. 2a,c,d). GSE treatment caused significant decrease both in the number of GST-p positive foci and in the area per cm^2^ compared to rats received the carcinogen alone (Fig. 2a,c,d).

### Effect of GSE on cell proliferation and apoptosis and HDAC activity in DEN-2AAF-treated rats

**Figure 3:**
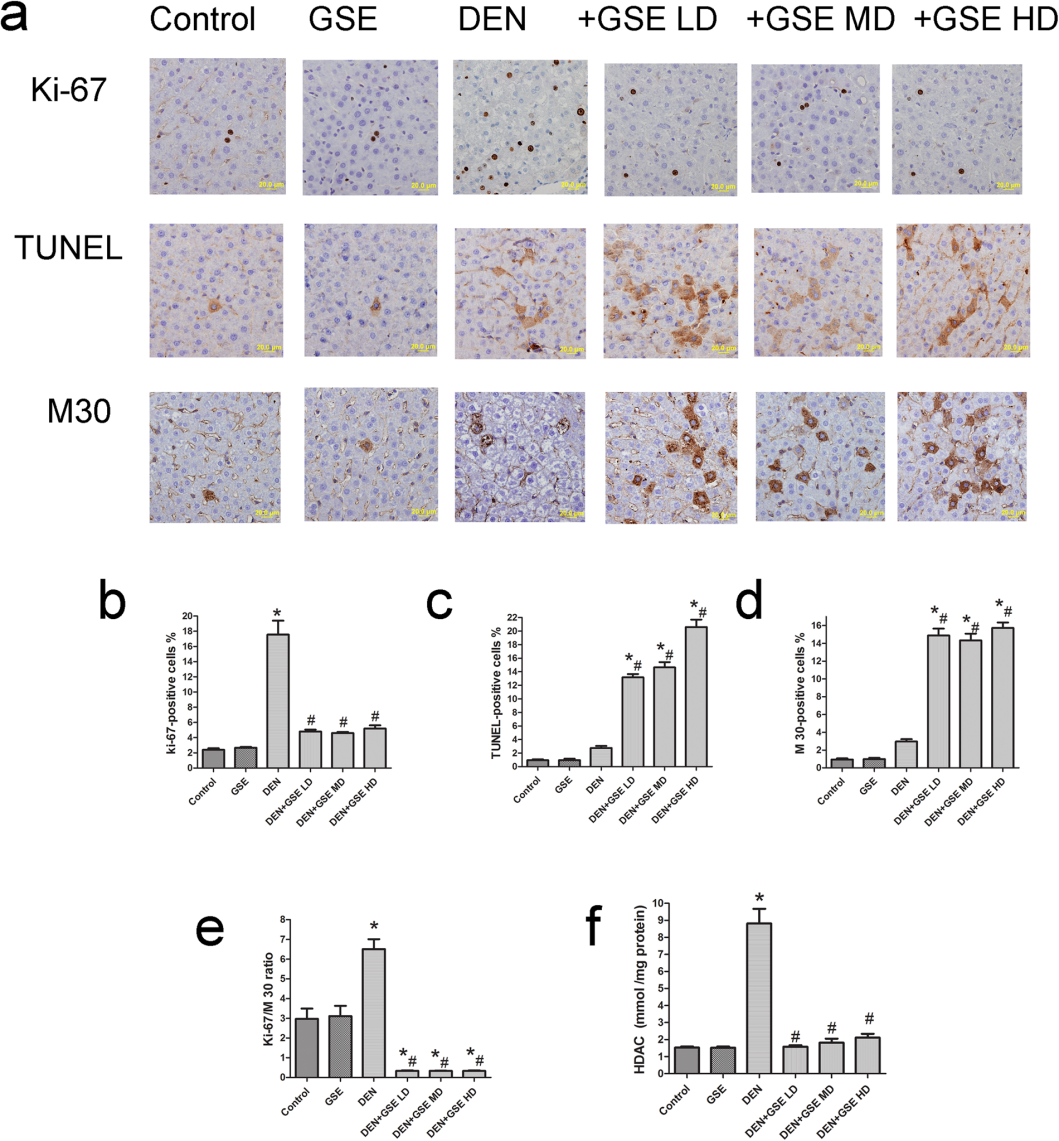
Effects of GSE on cell proliferation (Ki-67) and apoptotic cell death (TUNEL and with M30) and activity of HDAC. (**a**) The upper panel is representative images of immunohistochemical staining with Ki-67, TUNEL and M30 in the liver section from all the groups. (Scale bar = 20 µm). The lower panel shows and quantitative analysis of Ki-67 (**b**), TUNEL (**c**) and M30 (**d**) as well as Ki-76/M30 ratio (**e**) positive cells and HDAC activity (**f**), concentration is expressed in mmol/ mg protein, n = 6 in different experimental groups. The positive expression of Ki-67 (**b**), TUNEL (**c**) and M30 (**d**) in each section was calculated by counting the number of brown staining in ten fields at 400× magnifications, then, the number of each of ki-67, Tunnel and M30- positive cells was expressed as the number of positive hepatocytes ×100/total number of hepatocytes analyzed. Values expressed as mean ± s.e.m. for six animals in each group. Significance was determined by one-way analysis of variance followed by Dunnett’s t-test: *P < 0.05, vs. control group, ^#^P < 0.05 vs. DEN-2AAF group.

The nuclear Ki-67 is an established marker of cellular proliferation^22^. Liver sections of DEN-2AAF group showed significantly higher numbers of Ki-67 positive cells compared to the control group. Expectedly, compared to control, no significance was noted for the number of Ki-67 positive cells in liver sections from animals treated with GSE alone. Nevertheless, numbers of Ki-67-expressing cells in liver sections from DEN-2AAF -treated animals were significantly reduced upon treatment with different doses of GSE (Fig. 3a and b).

For determination of early apoptosis, M30 CytoDeath antibody was used as an apoptotic marker that specifically detects an epitope caused by caspase-cleaved keratin 18^23^. In addition, TUNEL assay was used to detect apoptosis-induced DNA fragmentation. Treatment with GSE alone did not induce significant increase in either of TUNEL-positive cells or M30 CytoDeath positive cells compared to Group 1. However, in liver sections of animals treated with DEN-2AAF the number of TUNEL-positive cells and M30 CytoDeath-positive cells were non-significantly higher in comparison to control animals (Fig. 3c and d). More importantly, the increased ratio between cell proliferation and apoptosis clearly indicated an imbalance between cell proliferation and cell death in liver of DEN-2AAF-treated rats. Nevertheless, the number of cells positive for TUNEL and M30 CytoDeath were significantly increased in groups treated with GSE and DEN-2AAF (Fig. 3c and d) where the ratio between cell proliferation and apoptosis decreased suggesting an up-regulation of apoptosis and down regulation of cell proliferation by GSE administrations in DEN-2AAF-induced rats (Fig. 3e). The effect of GSE on the activity levels of HDAC was evaluated *in vivo*. Livers of Group 2 animals exhibited no significant change in HDAC activity, compared to control group. Nonetheless, exposure to DEN-2AAFcaused a significant increase in HDAC activity, in comparison to control group. Pretreatment with GSE restored normal expression levels of HDAC in animals treated with different doses of GSE prior to HCC (Fig. 3f).

### GSE inhibited the upregulation of MPO activity and ED-1, ED-2, COX-2 & iNOS expression in liver of DEN-2AAF-treated rats

**Figure 4:**
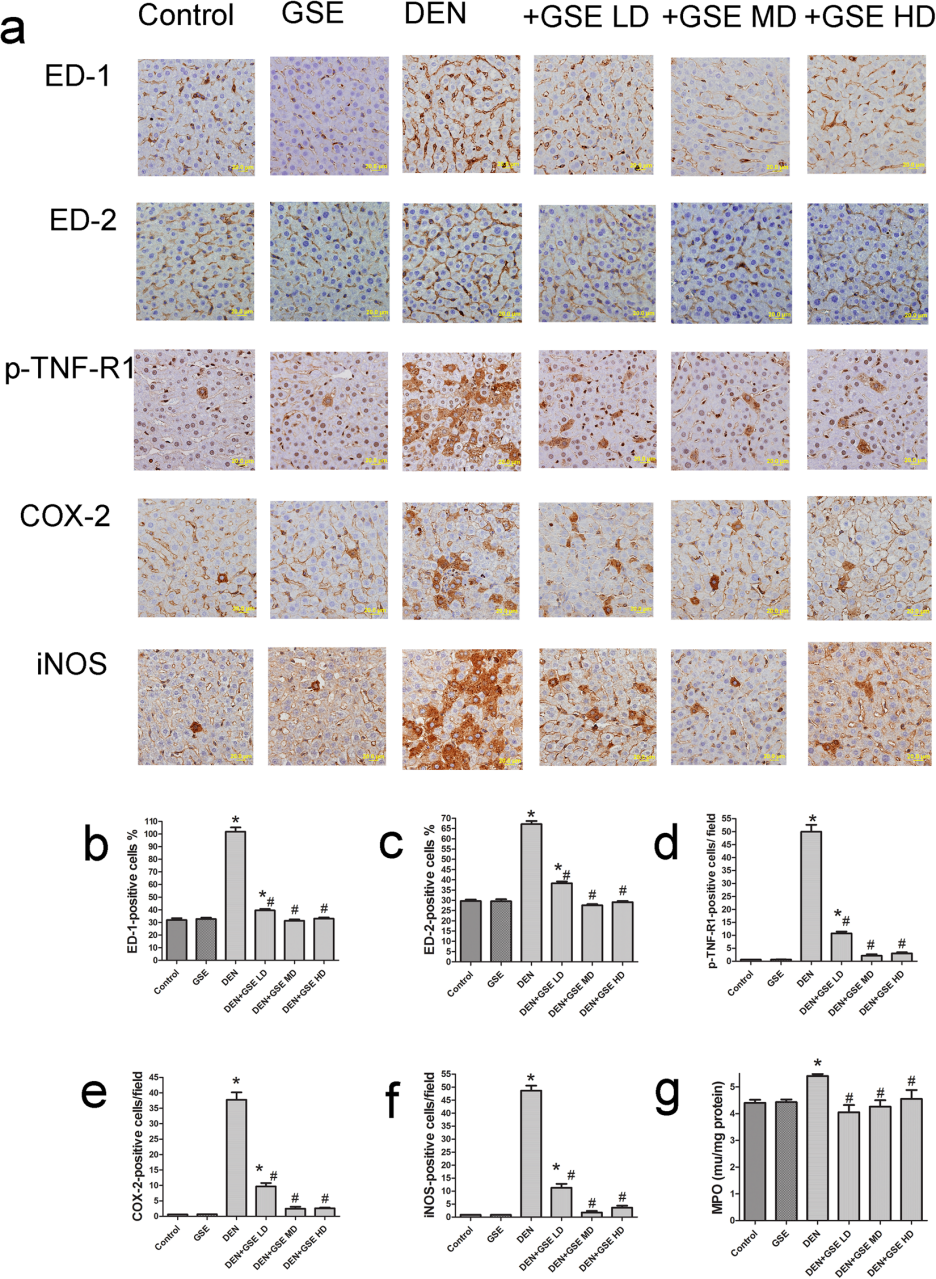
GSE inhibits DEN-2AAF-induced elevation in hepatic MPO activity and up regulation of ED-1, ED-2, p-TNF-R1, COX-2 and iNOS-positive cells expressions. The upper panel (a) is representative images of immunohistochemical staining with ED-1, ED-2, p-TNF-R1, COX-2 and iNOS in the liver section from all the groups. (Scale bar = 20 µm). (**b**–**g**) show quantitative analysis of ED-1, ED-2, p-TNF-R1, COX-2 and iNOS-positive cells as well as MPO activity. The positive expression of cells in each section was calculated by counting the number of brown staining in ten fields at 400× magnifications then the number of positive cells /field. The number of each ED1 and ED2- positive cells was expressed as the number of positive cells ×100/total number of hepatocytes analyzed. Data are represented as mean ± s.e.m. for six animals in each group. Significance was determined by one-way analysis of variance followed by Dunnett’s t-test: *P < 0.05 vs. control group, ^#^P < 0.05, vs. DEN group.

To assess macrophages’ level of activity, ED-1 was used as a cellular marker and to assess the activity of Kupffer cells resident macrophages, ED-2 was used as a cellular marker. In liver sections of DEN-2-AAF treated animals, a dramatic overexpression of ED-1 and ED2-expressing cells were evident (Fig. 4a,b and c). Such up-regulation of macrophages and Kupffer activities were significantly inhibited by treatment with GSE at different doses. No significant difference was detected in liver sections of animals in control Groups. Phospho tumor necrosis factor receptor 1 (p-TNF-R1) positive cells exhibited an increase in number in liver sections of animals treated with DEN-2-AAF (Fig. 4d). Nevertheless, the number p-TNF-R1 positive cells in liver sections from DEN-2-AAF-treated animals were significantly reduced in animals pretreated with different doses of GSE in comparison to that of the HCC-induced animals. Treatment with GSE alone had no significant effect on the number of p-TNF-R1 positive cells in comparison to the control groups. Additionally, DEN-2-AAF exposure significantly increased the expressions of cyclooxygenase (COX-2) and inducible nitric oxide synthase (iNOS), which were expressed mostly in hepatocytes around the central vein and in Kupffer’s cells (Fig. 4e and f). Those levels were significantly decreased upon pre-treatment with different doses of GSE compared to control animals. The effect of GSE, on the activity levels of MPO was evaluated in liver. Livers of GSE exhibited no significant change in MPO activity, compared to group 1. Nonetheless, DEN-2AAF-exposure caused an increase in the hepatic MPO activity (a marker of neutrophil infiltration), which was significantly decreased in GSE-treated groups compared to HCC group (Fig. 4g). Pretreatment with GSE restored normal activity of MPO in animals treated with low and high doses of GSE prior to HCC induction (Fig. 4g).

### GSE inhibited the expression and nuclear localization of NF-kB-p65 in liver of DEN-2AAF-treated rats

**Figure 5:**
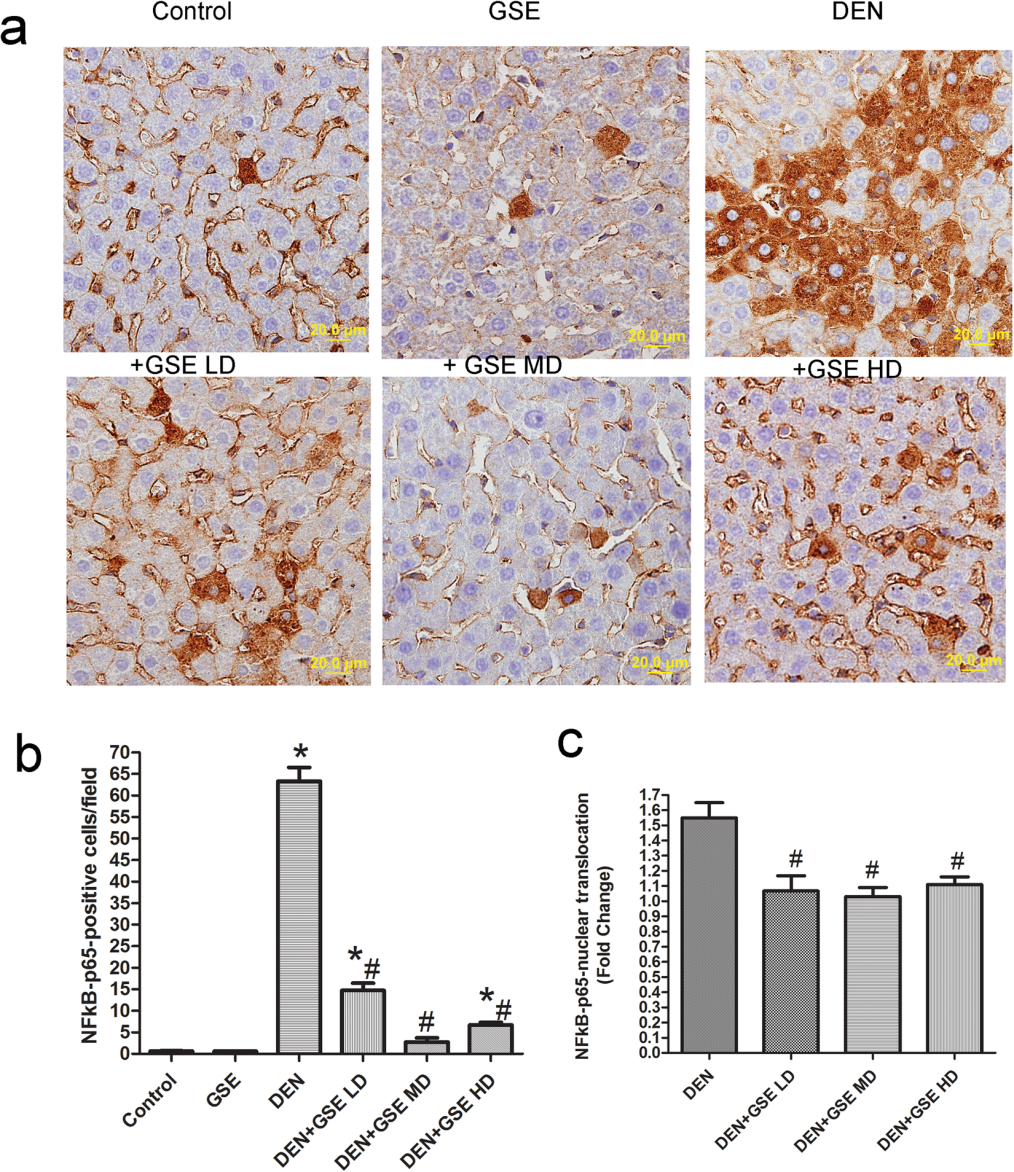
GSE inhibits DEN-2AAF-induced up regulation expressions and nuclear translocation of NF-_k_B- p65. The upper panel showing images of immunohistochemical NF-_k_B- p65- stained section in the livers of all groups studied (**a**). The lower panel shows quantitative analysis of NF-_k_B- p65 -positive cells (**b**) and its nuclear localization (**c**). The positive expression of NF-_k_B- p65 in each section was calculated by counting the number of brown staining in ten fields at 400× magnifications then the number of positive cells /field. NF-_k_B- p65 nuclear localization represented as fold change (relative to control group). Data are represented as mean ± s.e.m. for six animals in each group. Significance was determined by one-way analysis of variance followed by Dennett’s t-test: *P < 0.05 vs. control group, ^#^P < 0.05 vs. DEN-2-2AAF group.

DEN-2-AAF exposure significantly increased the expression of nuclear factor-kappa B p-65 (NF-_k_B-p65) positive cells, which were expressed mostly in hepatocytes around the central vein and in Kupffer’s cells (Fig. 5a). This increase in NF-_k_B-p65-positive cell numbers was significantly inhibited in GSE protected groups compared to rats that received DEN-2AAF alone (Fig. 5b). As parallel with the NF-_k_B-p65 expression, GSE treatment resulted in decreased level of NF-_k_B-p65 in nuclear extracts indicating that GSE is able to inhibit NF-_k_B translocation to the nucleus (Fig. 5c). This indicates that the anti-inflammatory effect of GSE in HCC model system could be due to blocking of the NF-_k_B signaling.

### Toxicity of GSE and DEN

During the experimental period only two animals from DEN-2AAF group died. In addition, there was no considerable difference food and water consumption between control and GSE-treated rats (data not shown). Supplementary Table 1 summarized data regarding the final body weight, liver weight, and relative liver weight of all animal groups after experimental period. There was a dramatic decrease in the final body weight of DEN-2AAF group. GSE administrations in DEN-2AAF-treated groups caused significant increase in the final body weight compared to rats received the carcinogen alone; however, those body weights remained significantly lower than control. The liver weight of DEN-2AAF group was significantly (p < 0.05) increased as compared with control rats. A similar observation was noted for the liver weights in animals treated with different doses of GSE prior to HCC. However, the relative liver weight was significantly (p < 0.05) lowered in DEN-2AAF + GSE treated rats when compared with HCC. There were no differences in all evaluated parameters among control and GSE-only rats. These observations suggest that the selected doses of GSE did not produce any apparent toxicity in the present study.

### GSE induced growth arrest and apoptosis *in vitro*

**Figure 6:**
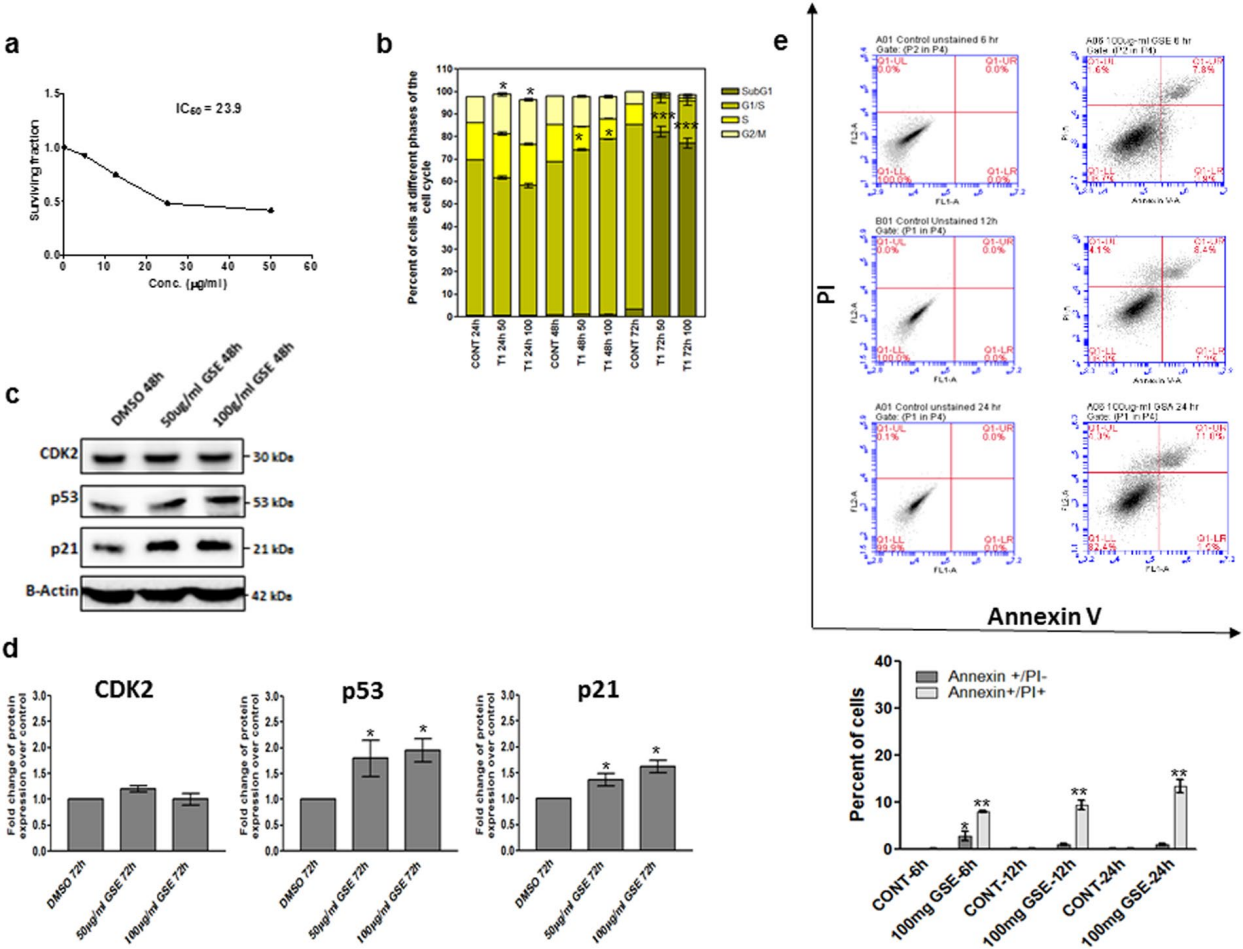
The effect of GSE on HepG2 (**a**) sulphorhoramine-B assay viability test. (**b**) Effect of GSE on cell cycle progression of HepG2 cells measured after treatment of the cells with GSE (50 and 100 µg/mL) for different time intervals. (**c**) Effect of GSE on the expression of proteins (CDK2, p53 and p21) involved in cell cycle regulation. (**d**) Quantification of proteins analyzed in panel (**c**), the band intensities were quantified using ChemiCoc Touch imaging from BioRad. β-actin served as an internal control for equal loading and data are performed in triplicates. (**e**) Induction of apoptosis in HepG2 cells after treatment with GSE measured by Annexin V staining (BD Biosciences). Annexin + /PI- represents cells undergoing early apoptosis whereas Annexin + /PI + represents late apoptotic and necrotic cells. Statistical analysis was carried out using Prism program (San Diego, USA). Significance of difference from the control cells is described as *P < 0.05; **P < 0.01: ***P < 0.001. Data are represented as mean ± s.e.m. Data shown are performed in triplicates.

To better understand the anticancer effects of GSE, more detailed *in vitro* analyses were carried out. HepG2 cells were treated with various concentrations of GSE (5, 12.5, 25 and 50 µg/mL) for 48 h. The SRB test showed that GSE significantly reduced the viability of HepG2 cells in a dose-dependent manner (Fig. 6a). GSE at a concentration of 23.9 µg/mL was able to reduce cell viability by almost 50% (Fig. 6a).

Effect of GSE on the cell cycle progression was assessed using the flow cytometry analysis. Figure 6b showed that GSE induced G2/M arrest by the significant increase of cells in G2/M phase (16.5% and 18.5%) after 24 h of treatment with 50 and 100 µg/ml GSE, respectively. G1/S arrest was also induced and manifested by significant increase of cells in G1/S phase (72.7% and 78%) after 48 h of treatment with 50 and 100 µg/ml GSE, respectively. In parallel, the percentages of sub-G1 phase (apoptotic cells) were significantly increased up to 72 and 77% after 72 h of treatment with 50 and 100 µg/mL of GSE, respectively (Fig. 6b). Through analyzing two key regulators of cell cycle arrest and apoptosis, namely p53 and p21, we confirmed the flow cytometry analysis. Western blot analysis showed that there was a significant up-regulation of p53 and p21 protein expression in HepG2 cells (Fig. 6c and d). Induction of apoptosis was also confirmed by detection of Annexin V staining of HepG2 cells after treatment with 100 µg/ml GSE. As shown in Fig. 6e, the percent of early apoptosis fraction (Annexin V positive/PI negative cells) was significantly increased to 3.8% after 6 h of treatment compared to control cells. The apoptosis fraction induction further increased with time reaching 11.8% after 24 h of treatment.

### Molecular mechanisms for GSE induced apoptosis in HepG2

**Figure 7:**
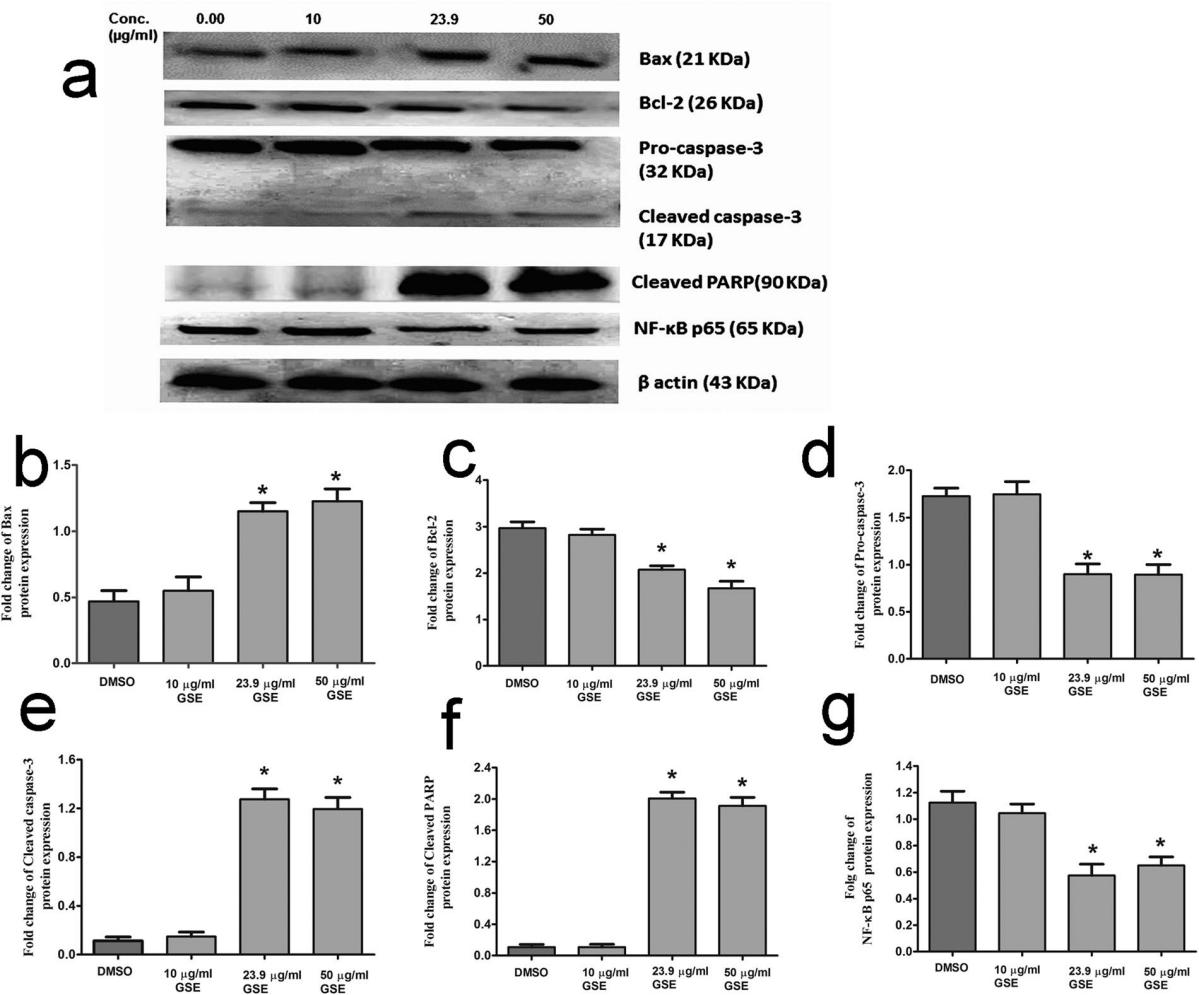
GSE extract induces apoptosis in liver cancer cell line HepG2. (**a**) Effect of GSE on the expressions of Bax (**b**), treated with DMSO (control) or treated with 10, 23.9 and 50 µg/mL GSE for 48 h, harvested, Lysates prepared from HepG2 cells treated with GSE for 48 h were analyzed by anti-BCL-2, Bax, Caspase 3, Procaspase 3, Cleaved PARP and anti-β actin western blotting. β-actin served as an internal control for equal loading (**a**) and data were quantified. Data are represented as mean ± s.e.m. Data shown are performed in triplicates. Significance of difference from the control cells is described as *P < 0.05.

To examine the effect of GSE on the apoptosis pathway, the protein level of Bcl-2-associated X protein (Bax) and B-cell lymphoma 2 (Bcl2), pro-caspase 3, cleaved caspase-3 and cleaved poly[ADP-ribose] polymerase (PARP) were analyzed by western blotting (Fig. 7a). In support of the previous *in vivo* results, HepG2 cells showed a remarkable reduction of Bcl-2 Fig. 7c and induction of Bax (Fig. 7b) 48 h after GSE’s administration thereby reflecting a strong pro-apoptotic effect of GSE. For further studies, GSE concentrations of 10, 23.9 and 50 µg/mL were administered. In support of the previous *in vivo* results, protein level of pro-caspase 3 was reduced 48 h after treatment with GSE and the effect was dose-dependent (Fig. 7d). In addition, the cleaved form of apoptotic proteins caspase-3 and PARP (Fig. 7e and f) were also significantly increased in dose-dependent manner. Finally, and in agreement with the *in vivo* results, GSE treatment reduced the NF-κB p65 protein expression (Fig. 7g).

## Discussion

This study investigated the protective effects of the herbal drug, GSE, on the early hepatic pre-neoplastic events, using a two-stage carcinogenic model combining DEN and 2AAF. GSE’s anti-oxidative stress, anti-inflammatory, anti-proliferative, and pro-apoptotic effects are well-documented here both *in vitro* and *in vivo*. We tested three doses of GSE; namely25, 50 and 100 mg/kg body weight. Administration of GSE to DEN-2AAF- treated rats reduced the development of pre-neoplastic FAH. This GSE-mediated reduction of FAH formation was closely associated with a significant decrease in the number of GST-P positive foci that represents a reliable and sensitive marker of pre-neoplasia and neoplasia and a well–documented end-point lesion in liver carcinogenicity^9,24^.

Persistent increase in cellular proliferation and disrupted apoptosis machinery are key alterations in the physiology of cancer cells that are essential for tumor progression^7,25,26^. In this study, GSE demonstrated a significant anti-proliferative activity in DEN-2AAF-treated animals by dramatic reduction of Ki-67 expression which has been shown to be associated with proliferations and to be present during all active phase of cell cycle (G1, S, G2 and mitosis)^22^. Further, in DEN-2AAF-treated rats, the anti-proliferative activity of GSE was also associated with the induction of apoptosis as evidenced by increasing the number of TUNEL- and M30 CytoDeath- positive cells. These results are in agreement with previous *in vitro* and *in vivo* studies reporting both apoptosis and anti-proliferative effects of GSE and its phenolic compounds in various tumor cell lines and in mice models of skin and mammary and prostate, and liver cancers^13–15,17,27^. These results indicate that the decrease of FAH formation and hepatic pre-neoplasia in rat was associated with a reduction of cell proliferation and an induction of apoptosis.

A similar trend was evident *in vitro* where a significant decrease in cell viability was observed in GSE-treated HepG2 cells where induced apoptosis was confirmed by detection of Annexin V staining of HepG2 cells. In addition, GSE caused significant increase in apoptotic proteins Bax, cleaved caspace-3 and cleaved PARP which was consistent with the GSE-induced apoptotic effect *in vivo*. Additionally, we investigated the effect of GSE on cell cycle progression. In the present study, GSE induced cell cycle arrest of the HepG2 cells by the accumulation of cells in G2/M phase and G1/S phase with concomitant increase in Sub G1 phase population. Several studies have demonstrated that GSE can induce both apoptosis and cell cycle arrest, for example GSE increased G2/M phase arrest of the cell cycle leading to induction of apoptosis in a dose- and time-dependent manner in pancreatic cancer cell^28^. GSE also induces inhibition of cell growth in human bladder cancer cells by arresting cell cycle at G1 phase and inducing cell apoptosis^12^. In parallel, we observed that GSE treatment resulted in an increase in the expression of p53 and p21. Since p53 –mediated induction of cell cycle arrest via upregulation of p21^29^, and induction of apoptosis via downregulation of Bcl2^30^, here we suggested that GSE could increase the accumulation of cells in G2/M phase and G1/S phase via a mechanism that incorporated increased expression of p21 and p53.

Most phenolic antioxidant compounds exhibit anti-proliferative activity and apoptosis in cancer cells^31^. Increased oxidative stress can induce a wide spectrum of cellular damage and cellular signaling changes that has been associated with carcinogenesis such as genomic instability and high cell proliferation, which contribute to FAH development and ultimately, to FAH progression to adenomas and HCC^32,33^. Administration of GSE to DEN-2AAF-treated rats in this work counteracted DEN-2AAF-induced oxidative stress as shown by the restoration of antioxidant levels of SOD and CAT in the liver and the diminishing of important markers of oxidative stress, such as MDA and P.Carbonyl. DEN-2AAF-induced CAT activity has been similarly shown to be increased in cancer cells, most likely, to favor cell proliferation by inducing genetic instability and activation of oncogenes^6,34^. The prevention of oxidative stress by GSE might be attributed to its potent antioxidant capacity that was confirmed in this study. GSE showed ABTS and DPPH radical scavenging activities and exhibited significant reducing power as indicated by the FRAP assay and prevented bleaching of ß-carotene. The Antioxidant property of GSE could be credited to its phenolic content and to its active ingredients including catechine, and epicatechin (Tables 1 and 2) which have been identified as the most potent antioxidant phenolic compounds of GSE^10,11^. The association between decreased oxidative damage and reduced GST-P positive foci formations suggest that the antioxidant efficacy exhibited by GSE may be an important factor for its anti-carcinogenic property. Most of plant polyphenols possess both antioxidants as well as prooxidants properties. Given their prooxidant capacity, plant polyphenols have been reported to induce DNA degradation and apoptosis in the presence of metal ions such as copper in cancer cells^35^. Our group along with others have reported that it was an endogenous copper-dependent prooxidants cytotoxic action of GSE rather than its antioxidant property that may contribute more to its anticancer and proapoptotic capacity in cancer cells^35,36^. This could explain the GSE apoptotic effect in cancer cells and protective effects in normal cells in the present study. Therefore, the chemopreventive activity of GSE derived polyphenols may be attributed to combination of their cytoprotective effects on normal cells (antioxidants-dependent mechanisms) and their cytotoxic effects on neoplastic cells (prooxidants- dependent mechanisms).

Chronic inflammation is known to trigger early changes associated with the development of cancer through activation of hepatic tumor associated-macrophages and Kupffer cells (mirrored by ED1, and ED2 respectively) as well as the production of pro-inflammatory mediators such as tumor necrosis factor-alpha (TNF-α), nitric oxide (NO) and transcription factors such as NF-_k_B^33,37–39^. In the present study, GSE displayed an efficacy to protect against DEN-2AAF-induced liver inflammation by decreasing numbers of ED1- and ED2- positive macrophages and levels of hepatic MPO, a marker of neutrophil infiltration^40^. This decrease in hepatic macrophages and neutrophils was accompanied by dramatic decrease in TNFR1-positive cells in liver. TNF-α is a major mediator of proliferations and inflammations and its tumor-promoting action involves TNFR1 activation^37^. Increased expressions of COX-2 and iNOS have been observed in several human tumor tissues and in chemically induced animal tumors^33,38^ and the chemopreventive effects of phytochemicals have been shown to be mediated with the inhibition of these enzymes^39,41^. In the present work, both COX-2 and iNOS levels were dramatically decreased in GSE-treated animals. The decrease of hepatic macrophages and neutrophils seems to be associated with an inhibited the protein expressions of COX-2 and iNOS – both of which are key enzymes involved in producing pro-inflammatory signals. Similarly, grape seed pro-anthocyanidin (GSP) modulates inflammatory response in activated macrophages by the suppression of iNOS expression and NF-kB translocation^42^. GSP inhibited mouse skin cancer that is associated with inhibition of COX-2 expression, leukocytes infiltrations and MPO activity in the mouse skin^15^.

Besides its properties as master regulators of inflammatory response in HCC, NF-κB is a redox-sensitive transcription factor present in the cytoplasm in its inactive form^33,43^. Under oxidative stress, NF-κB gets activated and translocated to nucleus where it enhances the expression of many genes involved in cell proliferation, cell death and inflammatory responses (as COX-2, iNOS and Bcl-2)^33^. Consistent with this view, DEN induced up-regulation and nuclear translocation of NF-κB-p65 subunit. Suppressing the activity of this transcription factors by phenolic rich extracts would be expected to thwart the ability of cancer cells to thrive^8^. Accordingly, the prevention of different NF-κB-related events, including the nuclear translocation of NF-κB subunits (i.e., p65), the DNA binding activity and the degradation of the inhibitor IkBα, by medicinal plants has been reported^44^. This notion was supported by present observations that administration of GSE to HCC-induced groups reversed DEN-2AAF-induced up-regulation and nuclear translocation of NF-κB-p65 subunit. Such results were supported by *in vitro* analyses where GSE-treated HepG2 cells demonstrated a reduction in NF-κB-p65 protein level. In previous study, GSP reduced the growth of skin cancer in nude mice through the suppression of NF-_k_B activity^15^. Taken together, these results suggest that GSE-based protection against carcinogenesis could be mediated by its anti-inflammatory activity through down regulating NF-κB, COX-2 and iNOS expressions levels and decreasing leukocytes infiltrations as well as the expressions of both TNFα and its receptors in tumor cells.

Another attractive explanation for chemopreventive effect offered by GSE is linked to the suppression of HDAC leading to transcriptional repression. HDAC enzymes remove acetyl groups from lysine amino acid on a histone leading to transcriptional repression^45^. HDAC upregulation is characteristic of many types of cancer and HDAC inhibitors are able to inhibit proliferation and inflammation and to trigger apoptosis in tumor cells^29,45^. In the present work, GSE demonstrated a significant efficacy in inhibiting the elevated HDAC activity in DEN-2AAF-treated rats. These results suggest the GSE’s anti-proliferative, anti-inflammatory and pro-apoptotic effects could be attributed, at least in part, to their ability to inhibit HDAC activity in cancerous cells. Therefore, HDAC inhibitory effect of GSE might be one of the chemopreventive effects of GSE. Consistently, Grape seed pro-anthocyanidins has been reported to decrease HDAC in human squamous cell carcinoma cell lines^46^. In this study, the chemopreventive properties of GSE was manifested through its ability to abrogate oxidative stress, inflammation and proliferation. Given the well-documented interaction between inflammation, oxidative stress and cancer^33^^,^^43^, phenolic compounds of GSE may contribute the most to its anti-carcinogenic property. Herein, GSE’s anti-proliferative, anti-inflammatory and pro-apoptotic effects could also be attributed, at least in part, to its antioxidant-mediated ability to inhibit NF-kB and HDAC activities. Interestingly, the effect of GSE was not dose-dependent. For the most part, the protective effect of GSE was most dramatic in rats exposed to the medium dose where it caused significant decrease in both the number and area of GST-p positive foci, and in all inflammatory markers.

**Figure 8:**
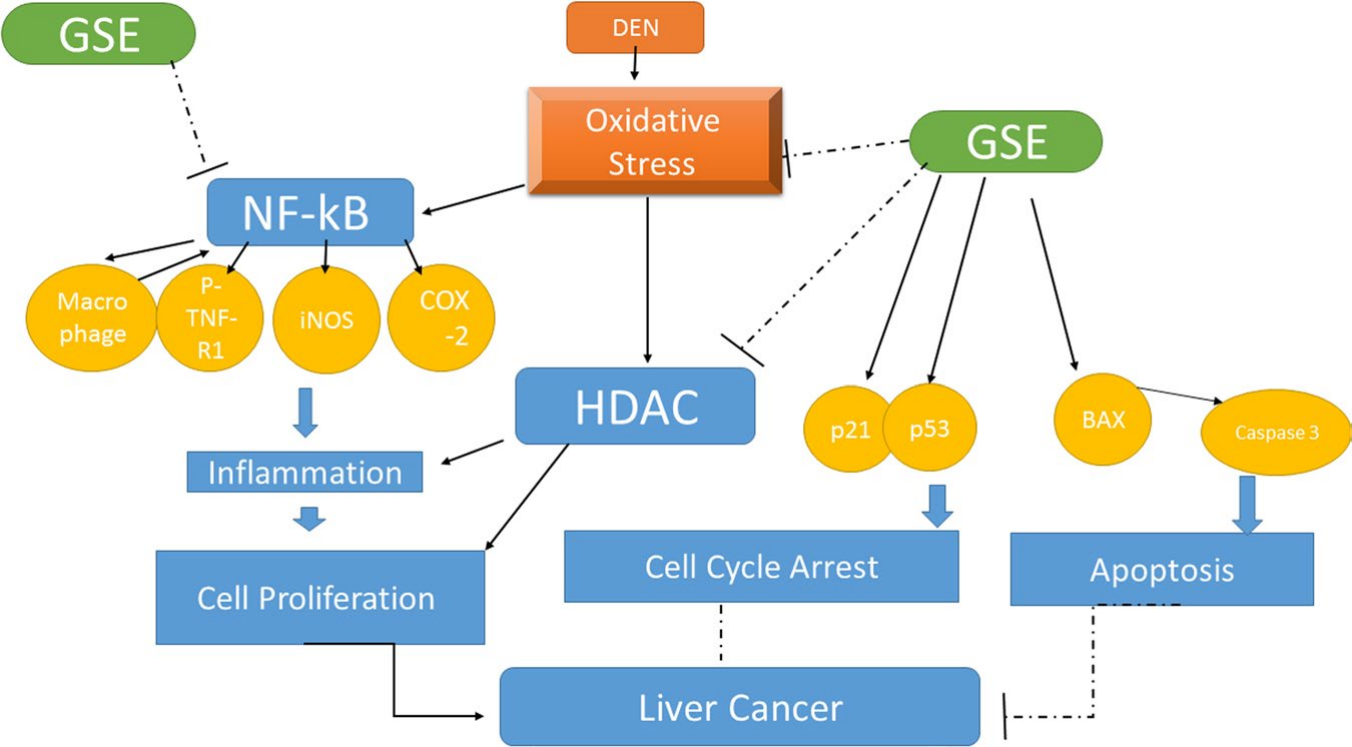
Schematic model showing the protective effect of GSE in DEN-2AAF-induced HCC by blocking proliferation and promoting apoptosis. This model incorporates all the *in vivo* data presented in this study. GSE prevents oxidative stress leading to attenuation of hepatic NF-_k_B and HDAC activations, which in turns leads to down regulation of NF-_k_B and HDAC signaling, including decrease in inflammation, proliferation and pro-apoptotic activities. GSE also causes cell-cycle arrest (stopping proliferation and inducing apoptosis).

In conclusion, the data presented here showed that GSE dramatically inhibited FAH formation in livers of DEN-2AAF-treated rats. GSE’s effects were associated with induced apoptosis, reduced cell proliferation, decreased oxidative stress and down regulation of HDAC activity and inflammation makers, such as COX-2, iNOS, NF-κB-p65 and p-TNFR1 expressions. Those effects are schematically depicted in Fig. 8, which incorporates our data into a model showing a possible mechanism of the anti-cancer protective effect of GSE by promoting apoptosis, inhibiting cell proliferation and blocking inflammation in hepatocarcinomas.

## Methods

### Animals

Adult male albino Wistar rats (150–200 g) were obtained from the Animal House, National Research Centre, Giza, Egypt. They were maintained on standard pellet diet and tap water *ad libitum* and were kept in polycarbonate cages with wood chip bedding under a 12 hrs. Light/dark cycle and room temperature 22–24 °C. Rats were acclimatized to the environment for two-weeks prior to experimental use. The protocol was conducted in accordance with standard guide to the care and use of experimental animals (Canadian Council of Animal Care 1993) and according to the ethical standards approved by the Institutional Animal Ethics Committee guidelines for animal care and use, Minia University, Egypt.

### Plant material and preparation

GSE capsule, 100 mg (01356US03SO) is standardized to polyphenols content (95%) and was donated from Solgar Vitamin and Herb (500 Willow Tree Road, N.J. 07605, USA, www.solgar.com). GSE capsule content was suspended in distilled water before application. This suspension was then used at a dose of 25, 50 and 100 mg/kg body weight in a volume of 5 ml/kg body weight.

### Phytochemical composition and *in vitro* antioxidant properties

Total phenolic, and total flavonoid contents as well as HPLC profile of individual polyphenols were determined as previously described^47^. An *in vitro* total antioxidant properties of GSE were estimated by the ferric reducing antioxidant power (FRAP), 1,1-diphenyl-2-picrylhydrazyl (DPPH·) and 2, 2-azino-bis(3-ethylbenzothiazoline-6-sulfonate) (ABTS·+) and ß-carotene bleaching tests. The FRAP assay is based on the reducing power of antioxidants in which a potential antioxidant will reduce the oxidized ferric ions to the ferrous ions^48^. ABTS assay is based on the reduction of blue green (ABTS·+) by antioxidants to its original colorless ABTS form^49^. DPPH assay is based on the reduction of DPPH· radical to a yellow colored compound^48^. Inhibition percentage = 100× (absorbance of the control - absorbance of the sample)/absorbance of the control. EC50 value (µg/mL) is the effective concentration at which DPPH· radicals are scavenged by 50% and is determined graphically. In ABTS and FRAP assays, calibration curves of ascorbic acid were established, the antioxidant capacity of the GSE was then expressed as mmol ascorbic acid equivalent/g dry extract. The ability of plant extract to prevent bleaching of ß-carotene was assessed as previously described^50^.

### Hepatocarcinogenesis model

The experimental hepatocarcinogenesis was initiated by DEN and promoted by 2-AAF, according to the protocol as previously described^21^ with some modifications. In this model, rats were injected intraperitoneally with a single dose of DEN (100 mg/kg b.wt.) dissolved in saline. After initiation, all rats underwent one period of 5-days fasting, as mitotic proliferative stimuli. Two weeks after DEN treatment, rats received 6 daily intragastric doses of 2-AAF then one dose every week for 4 weeks (30 mg/kg in 1% Tween 80), for promoting hepatocarcinogenesis,

### Treatment regime

Adult male Wistar rats weighing 180–210 g were obtained from the National Research Centre, Giza, Egypt. They were housed in clean polypropylene plastic cages and kept on a 12 h light/dark cycle and controlled temperature of 22 ± 2 °C. The animals were allowed to feed on standard chow pellets (supplemented with 18% proteins, 3% lipids and 10% fibers) and water *ad libitum*. The protocol was conducted in accordance with the ethical standards approved by the Institutional Animal Ethics Committee guidelines for animal care and use, Minia University, Egypt. Animals were left to acclimatize one week before starting the experiment, after which they were randomly divided into six groups (eight rats each). The experimental design is depicted in Fig. 1. Animals were subjected to the following treatments: Group 1 (Control): Rats were treated with distilled water (5 ml/kg b.wt.) throughout the experimental period and were injected with single dose of saline. Group 2 (GSE): Rats were administered orally 100 mg/kg GSE alone throughout the experimental period. Group 3 (DEN-2AAF-treated group): Rats were induced with DEN and 2-AAF as described before. Whereas animals of the protective groups (Groups 4–6) were orally fed 25 mg/kg (DEN-2AAF + GSE LD), 50 mg/kg (DEN-2AAF + GSE MD), and 100 mg/kg (DEN-2AAF + GSE HD), GSE suspension, respectively, at the beginning of promotion periods and continued for 12 weeks. It has been recognized that the inhibition of tumor promotion is most probably a better strategy for cancer chemoprevention than inhibition of tumor initiation stage because initiation is a short irreversible event, whereas the tumor promotion is reversible during the early stages^51^.

Doses of GSE were selected based on previously reported pharmacological studies of this plant. GSE at doses varying from 50 to 200 mg/kg has been reported to suppress chemically induced colon and prostate cancer^13,15^. No adverse effect has been reported for GSE up to 400 mg/kg treatment^48^. Hepatocarcinogenesis was then induced as detailed in the previous section. Control was treated with equal volume of vehicle. After 14 weeks of DEN administration, all animals were anesthetized by diethyl ether 24 h post last treatment. Blood samples were collected through retro orbital plexus and the animal sacrificed by cervical dislocation. The experimental design is illustrated in Fig. 1 and the effect of GSE on animals’ body weight and liver weight is shown in Supplementary Table 1.

### Morphology and Histopathology

Livers were excised. Samples of right, left and caudate lobes of livers were immediately fixed in 10% buffered formalin, for histopathological examination. The remaining liver was frozen in liquid nitrogen and stored at −80 °C. Histological sections were embedded in paraffin after being dehydrated in ethanol. Five-micrometer sections were mounted onto slides, stained with Hematoxylin and Eosin and were finally examined under Olympus DP71-light microscope, (Tokyo, Japan).

### Sample preparations

Liver homogenates were prepared by homogenization frozen liver samples in ice-cold Tris-HCL buffer (150 mM, pH 7.4), of 1:10 wt/v ratio. Aliquots were prepared for the determination of different biochemical markers.

### *In vivo* antioxidant status of liver

Determination of MDA in liver homogenate was carried out based on its reaction with thiobarbituric acid (TBA) to form a pink complex with absorption maximum at 535 nm as previously described^52^.

Protein carbonyl (P.carbonyl) contents were determined as previously described^53^. This method is based on spectrophotometric detection of the end product of reaction of 2, 4-dinitophenylhydrazine with P.carbonyl to form protein hydrazones at 370 nm. The results were expressed as nmol of carbonyl group per milligram of protein with molar extinction coefficient of 22000 M/cm.

The activity of myeloperoxidase (MPO) in homogenate was determined using the method reported by Hillefass *et al*.^54^. One unit of MPO was defined as the amount of MPO present that degrades one μM peroxide per a minute.

The activity of Catalase (CAT) activity was determined according to the method reported by Aebi^55^ by measuring the exponential disappearance of hydrogen peroxide (H_2_O_2_) at 240 nm and expressed in units/mg of protein.

The activity of superoxide dismutase (SOD) activity in liver homogenate was determined according to the method described by Nandi & Chatterjee^56^. This method is based on the ability of SOD to inhibit the auto-oxidation of pyrogallol at alkaline pH. One unit of SOD is described as the amount of enzyme required to cause 50% inhibition of pyrogallol auto-oxidation. The total protein contents of liver tissues were determined according to the Lowry method as modified by Peterson^57^.

### TUNEL assay and immunohistochemical analysis

To examine apoptotic cells, four-micrometer liver sections were prepared. They were subjected to be de-paraffinization and gradual hydration. Apoptotic bodies were detected by terminal deoxynucleotidyl transferase (TdT) mediated dUTP nick ending labeling (TUNEL) of DNA fragmentation using an ApopTag peroxidases *in Situ* Apoptosis Detection kit obtained from Serologicals Corporation (Norcross, GA, USA), according to the protocol supplied with the kit. Cell death was further confirmed by staining with M30 CytoDeath monoclonal antibody (Enzo life Science, Lausen, Switzerland).

For the proposes of immunohistochemical analysis, mounted sections were immersed in sodium citrate buffer (0.1 M, pH 6) and placed in a water bath for 15 minutes to unmask antigen epitopes. Then, endogenous peroxidase activity was blocked with 0.3% H2O2 in methanol. Rabbit anti-rat primary antibodies of Ki-67, anti-COX-2 (Clone SP 21), anti-iNOS (Ab-1), and anti-NF-kB-P65 (Rel A, Ab-1) were obtained from Thermos Fisher Scientific (Anatomical Pathology, Fremont, USA, 1:100 dilutions). In addition, monoclonal ED-1 anti-rat (CD 68, 1:300), and monoclonal ED-2 anti-rat antibody (CD 163, 1:300) and the phosphorylated form of tumor necrosis factor alpha receptor 1 (p-TNFR, polyclonal anti-rabbit antibody, 1:200) were purchased from Santa Cruz Biotechnology (CA, USA). Polyclonal anti-rabbit GST-p form was obtained from (Medical and Biological Laboratories Co., Tokyo, Japan, clone 311, 1: 1000 dilutions). Primary antibodies anti-GST-p, anti-Ki-67, M30 CytoDeath (Enzo life Science, USA), anti- COX-2, anti- iNOS, anti-NF-kB-p65 unit, anti-p-TNFR and anti-ED-2 were incubated with slides overnight at 4 °C. After washing the slides with phosphate buffered saline, sections were incubated with polyvalent biotinylated goat anti-rabbit secondary antibody diluted 1: 200 at room temperature for 10 min. Universal LSAB kit and DAB plus substrate kit were both used to perform a standard staining protocol and an additional counter-staining was performed using hematoxylin. Tissue images were captured by optical microscopy (Olympus DP71, Olympus, and Tokyo, Japan). Then, positive cells were quantified in ten randomly selected fields (magnification 400×) per individual samples. The number of ki-67, Tunnel and M30- positive cells was expressed as the number of positive hepatocytes ×100/total number of hepatocytes analyzed. The number of ED-1 and ED-2 positive cells was expressed as the number of positive cells X 100 total number of hepatocytes analyzed. GST-p foci larger than 15 cells were measure using color image processor. The number and areas (mm^2^) of foci /cm2 of liver sections were calculated.

### NF-kB nuclear localization assay

After nuclear extraction, the nuclear localized NF-kB was quantified using a transcription factor assay kit (10007889, Cayman Chemical Company, Ann Arbor, MI, USA) to detect activated p65 subunit of NF-kB, according to the manufacturer, s protocol.

### Histone deacetylase activity

HDAC activity in liver homogenate was measured with HDAC Colorimetric Assay Kit (Millipore Corporation, 28820 Single Oak Drive, Temecula, CA 92590, Catalog number: 17–374).

### *In vitro* studies

#### Cell culture

Cell culture work was done in Pharmacology Unit, Cancer Biology Department, National Cancer Institute, Cairo University, Cairo, Egypt. The cytotoxic effect of the tested drug was evaluated using human liver carcinoma cell line (HepG2). HepG2 was obtained frozen in liquid nitrogen from American Type Culture Collection (ATCC). Cells were grown as “monolayer culture” in maintained in Roswell Park Memorial Institute-160 medium with 10% fetal bovine serum and 1% of 100 U/mL penicillin and 100 µg/mL streptomycin at 37 °C inside a humidified incubator with 5% CO2 and 95% room air.

#### Cytotoxic effect of GSE *in vitro* study

Cytotoxicity was determined using Sulphorhodamine-B (SRB) method^58^. HepG2 cells were seeded in 96-well plates at the density of 10,000 cells/well and grown in 100 µL of complete growth medium, cells were allowed to attach for 24 h. After 24 h, media was replaced with new media supplemented with appropriate GSE concentrations. Different concentrations of the GSE (0, 5, 12.5, 25 and 50 μg/ml) were added to the cell monolayer for 48 h at 37 °C and triplicate wells were prepared for each individual dose. Dimethyl sulphoxide (DMSO) was used to dissolve the tested drug and then diluted thousand times in the assay. After treatment, cells were fixed with 10% trichloroacetic acid for 1 h at 4 °C.Wells were washed with water and then stained with 50 μl 0.4% SRB in 1% acetic acid for 30 min at 25 °C. The dye was solubilized with 100 μl 10 mM trizma® base (pH 10.5). Optical density of each well was measured spectrophotometrically at 564 nm. The relation between surviving fraction and drug concentration is plotted to get the survival curve of the cells and IC_50_ value (the concentration at which 50% of cell growth is inhibited) was calculated (GraphPad Prism software, version 5).

#### Cell cycle analysis

Changes in cell cycle distribution was analyzed by flow cytometry as previously described^59^. Cells were plated at a density of 5 × 10^6^ cells/ml in 75 cm^2^ culture flasks. After 24 h, the medium was replaced with fresh medium containing 50 or 100 µg GSE. Control cells were treated with the vehicle (DMSO) and all flasks were incubated at 37 °C. Cells were harvested by trypsin after different time intervals 24, 48 and 72 h, washed twice with phosphate buffered saline (PBS), fixed in 70% ethanol followed by treatment with Ribonuclease (RNAse). Fifty µl propidium iodide solution (1 mg/ml) was added and the fluorescence was measured with a FACS can flow cytometer (Becton Dickenson, Germany).

#### AnnexinV apoptotic assay

Induction of apoptosis by GSE was determined by measuring the percent of annexin V positive cells as previously described^60^. Cells were seeded in 75 cm2 culture flasks and treated after 18 h with 100 µg/ml GSE (or vehicle, DMSO). After different time intervals (6, 12 and 24 h), cells were detached and prepared as recommended by the FITC Annexin V Apoptosis Detection Kit (BD Biosciences). 5 µl of FITC Annexin V and 10 µl Propidium Iodide (500 µg/ml) were added and samples were incubated for 15 min in the dark at room temperature. Binding Buffer (400 µl) was then added. FACS can flow cytometer (Becton Dickenson, Germany) was used to assess the cell apoptosis.

#### Western Blotting

HepG2 cells were seeded in 100 mm plates (2 × 10^6^) in complete growth medium, and were allowed to attach for 24 h, prior to replacing their complete growth medium with serum-free medium. Cells were treated with GSE at a dose of (10, 23.9 and 50 µg/ml) and were incubated 48 h, in a humidified 5% CO_2_ atmosphere at 37 °C. After treatment, the cells were collected, washed with cold PBS and lysed. The protein content in the lysates was measured by BCA protein assay (Pierce, Rockford, IL, USA), according to protocol of the manufacturer. Western blot analysis was carried out as previously described^61^. Briefly, aliquots of the total cell lysates were boiled for 5 min in sodium dodecyl sulfate polyacrylamide gel electrophoresis and equal amounts of protein (40 µg) were resolved on 10% polyacrylamide gel and transferred to polyvinylidene fluoride (PVDF) membranes. After transfer, the membranes were incubated with primary antibody against tested proteins (Bax and BCL2, pro-caspase 3, cleaved caspase-3 and PARP, Thermos Fisher Scientific, Fremont, USA, 1:2000 dilutions), NF-κB and p 53 and p 21, CDK2 and β-actin Santa Cruz Biotechnology CA, USA, 1:2000 dilutions), followed by incubation with a secondary horseradish peroxidase–conjugated antibody (Santa Cruz Biotechnology CA, USA, 1:3000 dilutions). The signals were visualized with enhanced chemiluminescent (ECL) substrates (Pierce, Rockford, IL, USA) and captured by a Versadoc imager system, Biorad, USA. Quantification of the proteins will be performed using the ImageJ software with β-actin as the inner control.

#### Statistical Analysis

Results are represented as mean ± s.e.m. SPSS (version 20) statistical program (SPSS Inc., Chicago, IL, USA) was used to carry out a one-way analysis of variance (ANOVA) on our data. When significant differences by ANOVA

were detected, analysis of differences between the means of the treated and control groups were performed by using Dunnett’s t-test. Figures were done using GraphPad Prism program (version 5) (San Diego, USA).

### Data availability

The authors declare that all data supporting the findings of this study are available within the paper and its associated files. All relevant data are available from the authors upon request.

## Electronic supplementary material

Supplementary information

## References

[1] Torre LA (2015). Global cancer statistics, 2012. CA: a Cancer J. Clinic..

[2] De Martel C (2012). Global burden of cancers attributable to infections in 2008: a review and synthetic analysis. The lancet oncology.

[3] Mittal S, El-Serag HB (2013). Epidemiology of HCC: Consider the Population. J Clin Gastroenterol..

[4] Park D-H (2009). Diethylnitrosamine (DEN) induces irreversible hepatocellular carcinogenesis through overexpression of G 1/S-phase regulatory proteins in rat. Toxicol. Lett..

[5] Hodek P (2009). Chemopreventive compounds-View from the other side. Chem. Bio.Inter..

[6] Amin A (2016). Saffron-based crocin prevents early lesions of liver cancer: *in vivo*, *in vitro* and network analyses. Recent pat. anti-can. drug disc..

[7] Maru GB, Hudlikar RR, Kumar G, Gandhi K, Mahimkar MB (2016). Understanding the molecular mechanisms of cancer prevention by dietary phytochemicals: From experimental models to clinical trials. World J. Bio. Chem..

[8] Neergheen VS, Bahorun T, Taylor EW, Jen L-S, Aruoma OI (2010). Targeting specific cell signaling transduction pathways by dietary and medicinal phytochemicals in cancer chemoprevention. Toxicology.

[9] Feo, F., Pascale, R. M. & Calvisi, D. F. Models for liver cancer. in *The* Cance *Handbook* (ed. Alison, M.R.) 1-12 (John Wiley & Sons, Ltd., 2007).

[10] Rababah TM, Hettiarachchy NS, Horax R (2004). Total phenolics and antioxidant activities of fenugreek, green tea,grape seed, ginger, rosmary,gotu kola, and ginkgo extracts, vitamin E and tert-butylhydroquinone. J. Agri. Food Chem..

[11] Nassiri-Asl M, Hosseinzadeh H (2009). Review of the pharmacological effects of *Vitis vinifera* (grape) and its bioactive compounds. Phytother. Res..

[12] Liu W (2016). Grape seed proanthocyanidin extract protects against perfluorooctanoic acid-induced hepatotoxicity by attenuating inflammatory response, oxidative stress and apoptosis in mice. Toxicol. Res..

[13] Raina K, Singh RP, Agarwal R, Agarwal C (2007). Oral grape seed extract inhibits prostate tumor growth and progression in TRAMP mice. Cancer Res..

[14] Kaur M, Agarwal C, Agarwal R (2009). Anticancer and cancer chemopreventive potential of grape seed extract and other geape-based products. The J. Nutr..

[15] Meeran SM, Vaid M, Punathil T, Katiyar SK (2009). Dietary grape seed proanthocyanidins inhibit 12-O-tetradecanoyl phorbol-13-acetate-caused skin tumor promotion in 7,12-dimethylbenz(a) anthracene-initiated mouse skin, which is associated with the inhibition of inflammatory responses. Carcinogenesis.

[16] Hamza AH, Abdulfattah HM, Mahmoud RH, Khalil WK, Ahmed HH (2015). Current concepts in pathophysiology and management of hepatocellular carcinoma. Acta Biochimica. Polonica.

[17] Sherif AA, Abdelhalim SZ, Salim EI (2017). Immunohistochemical and biochemical alterations following administration of proanthocyanidin extract in rats hepatocellular carcinoma. Biomed. Pharmacother..

[18] Li X, Zhou XP, Guan YS, Wang YX (2005). Msgnetic resonance imaging of hepatocellular carcinoma induced by diethylnitrosamine in Sprague-Dawley rats. Hepatobiliary Pancreat Dis Int..

[19] Santos NP, Colaço AA, Oliveira PA (2017). Animal models as a tool in hepatocellular carcinoma research: A Review. Tumor Biol..

[20] De Minicis S (2013). Liver carcinogenesis: rodent models of hepatocarcinoma and cholangiocarcinoma. Dig. Liver Dis..

[21] Espandiari P, Robertson LW, Srinivasan C, Glauert HP (2005). Comparison of different initiation protocols in the resistant hepatocyte model. Toxicology.

[22] Scholzen T, Gerdes J (2000). The Ki-67 protein: From the known and unknown. J Cellul.Physiol..

[23] Carr NJ (2000). M30 expression demonstrates apoptotic cell, correlates with *in situ* end-labeling and is associated with Ki-67 expression in large intestinal neopasms. Arch Pathol.Lab.Med..

[24] Tew KD (2011). The role of glutathione S-transferase P in signaling pathways and S-glutathionylation in cancer. Free Rad.Biol. and Med..

[25] Block, K.I., *et al*. Designing a broad-spectrum integrative approach for cancer prevention and treatment. *In Sem*. *in cancer Bio*., Vol. 35, S276–S304 (Elsevier, 2015). 10.1016/j.semcancer.2015.09.007 PMC4819002 26590477

[26] Kimura M (2016). Onset of hepatocarcinogen‐specific cell proliferation and cell cycle aberration during the early stage of repeated hepatocarcinogen administration in rats. J. Appl. Toxicol..

[27] Mantena SK, Baliga M, Katiyar SK (2006). Grape seed proanthocyanidins induce apoptosis and inhibit metastasis of highly metastatic breast carcinoma cells. Carcinogenesis.

[28] Prasad R, Vaid M, Katiyar SK (2012). Grape proanthocyanidin inhibit pancreatic cancer cell growth *in vitro* and *in vivo* through induction of apoptosis and by targeting the PI3K/Akt pathway. PloS one.

[29] Karimian A, Ahmadi Y, Yousefi B (2016). Multiple functions of p21 in cell cycle, apoptosis and transcriptional regulation after DNA damage. DNA repair.

[30] Dashzeveg N, Yoshida K (2015). Cell death decision by p53 via control of the mitochondrial membrane. Cancer Let..

[31] Roleira FM (2015). Plant derived and dietary phenolic antioxidants: anticancer properties. Food Chem..

[32] Valko M, Rhodes CJ, Moncol J, Izakovic M, Mazur M (2006). Free radicals, metals and antioxidants in oxidative stress-induced cancer. Chem.Biol.Inter..

[33] Reuter S, Gupta SC, Chaturvedi MM, Aggarwal BB (2010). Oxidative stress, inflammation, and cancer: How are they linked. Free Rad. Biol. Med..

[34] Glorieux C, Calderon PB (2017). Catalase, a remarkable enzyme: targeting the oldest antioxidant enzyme to find a new cancer treatment approach. Biol Chem..

[35] Khan HY, Zubair H, Ullah MF, Ahmad A, Hadi SM (2012). A Prooxidant mechanism for the anticancer and chemopreventive properties ofplant polyphenols. Curr. Drug Targets.

[36] Hamza AA, Ahmed MM, Elwey HM, Amin A (2016). Melissa officinalis Protects against Doxorubicin-Induced Cardiotoxicity in Rats and Potentiates Its Anticancer Activity on MCF-7 Cells. PloS one.

[37] Roberts RA (2007). Role of the Kupffer cell in mediating hepatic toxicity and carcinogenesis. Toxicol.Sci..

[38] Kundu JK, Surh YJ (2005). Breaking the relay in deregulated cellular signal transduction as a rationale for chemoprevention with anti-inflammatory phytochemicals. Mutat. Res..

[39] Gupta SC (2014). Downregulation of tumor necrosis factor and other proinflammatory biomarkers by polyphenols. Arch. Biochem. Biophy..

[40] Loria V, Dato I, Graziani F, Biasucci LM (2008). Myloperoxidase: A new biomarker of inflammation in ischemic heart disease and acute coronary syndromes. Mediators Inflamm..

[41] Kern M, Schirmacher P, Breinig M (2006). [Significance of cyclooxygenase-2 as a chemotherapeutic target in hepatocellular carcinoma]. Verhandlungen der Deutschen Gesellschaft fur Pathologie.

[42] Terra X (2007). Grape-seed procyanidins act as antiinflammatory agents on endotoxin-stimulated RAW 364.7 macrophages by inhibiting NFkB signaling pathway. J. Agricult. Food Chem..

[43] Luedde T, Schwabe RF (2011). NF-κB in the liver—linking injury, fibrosis and hepatocellular carcinoma. Natu. Rev. Gastroenterol. Hepatol..

[44] Cerella C, Sobolewski C, Dicato M, Diederich M (2010). Targeting COX-2 expression by natural compounds: a promising alternative strategy to synthetic COX-2 inhibitors for cancer chemoprevention and therapy. Biochem. Pharmacol..

[45] Walkinshaw, Yang (2008). Histone deacetylase inhibitors as novel anticancer theraputics. Current Oncol..

[46] Vaid M, Prasad R, Singh T, Jones V, Katiyar SK (2012). Grape seed proanthocyanidins reactivate silenced tumor suppressor genes in human skin cancer cells by targeting epigenetic regulators. Toxicol.Appl. Pharmacol..

[47] Muanda F, Kone D, Dicko A, Soulimani R, Younos C (2009). Phytochemical composition and antioxidant capacity of three Malian medicinal plant parts. eCAM September.

[48] Nenadis N, Lazaridou O, Tsimidou MZ (2007). Use of reference compounds in antioxidant activity assessment. J.Agric.Food.Chem..

[49] Erel O (2004). A novel automated direct measuremnet method for total antioxidant capacity using a new gemeration, more stable ABTS radical cation. Cli. Biochemistry..

[50] Lim YY, Quah EPL (2007). Antioxidant properties of different cultivars of *Portulaca oleracea*. Food Chem..

[51] Meeran SM, Katiyar SK (2008). Proanthocyanidins inhibit mitogenic and survival-signaling *in vitro* and tumor growth *in vivo*. Front Biosci.

[52] Uchiyama M, Mihara M (1978). Determination of malonaldehyde precursor in tissues by thiobarbituric acid test. Anal Biochem.

[53] Reznick AZ, Packer L (1994). Oxidative damage to proteins:Spectrophotometric method for carbonyl assay. Metho.Enzymol..

[54] Hillefass L, Griswold D, Brickson B, Albrightson-Winslow C (1990). Assessment of myeloperoxidase activity in whole rat kidney. J. Pharm. Method..

[55] Aebi H (1984). [13] Catalase *in vitro*. Methods Enzymol.

[56] Nandi A, Chatterjee IB (1988). Assay of superoxide dismutase activity in animal tissues. J Biosci.

[57] Peterson GL (1977). A simplification of the protein assay method of lowry *et al* which is more generally applicable. Anol. Biochem..

[58] Skehan P (1990). New colorimetric cytotoxicity assay for anticancer-drug screening. J. Natio. Cancer Instit..

[59] Saleh EM, El-awady RA, Eissa NA, Abdel-Rahman WM (2012). Antagonism between curcumin and the topoisomerase II inhibitor etoposide: a study of DNA damage, cell cycle regulation and death pathways. Cancer Biology. Therapy..

[60] El-Awady RA (2016). Modulation of DNA damage response and induction of apoptosis mediates synergism between doxorubicin and a new imidazopyridine derivative in breast and lung cancer cells. DNA repair.

[61] El-Kady AI, Sun Y, Li Y-X, Lia DJ (2011). Cyclin D1 inhibits whereas c-Myc enhances the cytotoxicity of cisplatin in mouse pancreatic cancer cells via regulation of several members of the NF-κB and Bcl-2 families. J. Carcinog..

