# Supplementary material for: Molecular characterization of the grape seeds extract’s effect against chemically induced liver cancer: In vivo and in vitro analyses
Source: Sci Rep. 2018 Jan 19;8:1270. doi: 10.1038/s41598-018-19492-x (PMC5775207; doi:10.1038/s41598-018-19492-x)
Supplement: Supplementary file 1 — Supplementary information [file 41598_2018_19492_MOESM1_ESM.doc]

***Supplementary Information***

**Molecular characterization of the grape seeds extract’s effect against chemically induced liver cancer: *In vivo and in vitro* analyses**

**Alaaeldin Ahmed Hamza^1*^, Gehan Hussein Heeba^2^, Hanan Mohamed Elwy^3^, Chandraprabha Kumari^4^, Raafat El-Awady^5^**

**and Amr Amin^†4^,**

*1 Hormone Evaluation Department, National Organization for Drug Control and Research, Giza, Egypt*

*2 Department of Pharmacology and Toxicology, Faculty of Pharmacy, Minia University, El-Minia, Egypt*

*3 Analytical Chemistry Department, NODCAR, Giza, Egypt*

*4 Biology Department, UAE University, UAE*

*5 Department of Pharmacy Practice & Pharmacotherapeutics, University of Sharjah, UAE*

*6 Zoology Department, Cairo University, Egypt*

**corresponding to: Alaaeldin A .Hamza*

*E-mail:alaa17mm@gmail.com*

*NODCAR, 6 Abu Hazem St., Pyramids, Giza, Egypt-*

*Tel. 20.01201991433- Fax: ‎+20.2.33933897*

*† corresponding to: Amr Amin*

*^E-Mail^:* [*a.amin@uaeu.ac.ae*](mailto:a.amin@uaeu.ac.ae)

*Biology Department, UAE University, UAE*

*Tel .97137136519 – Fax: 9713-713492*

**
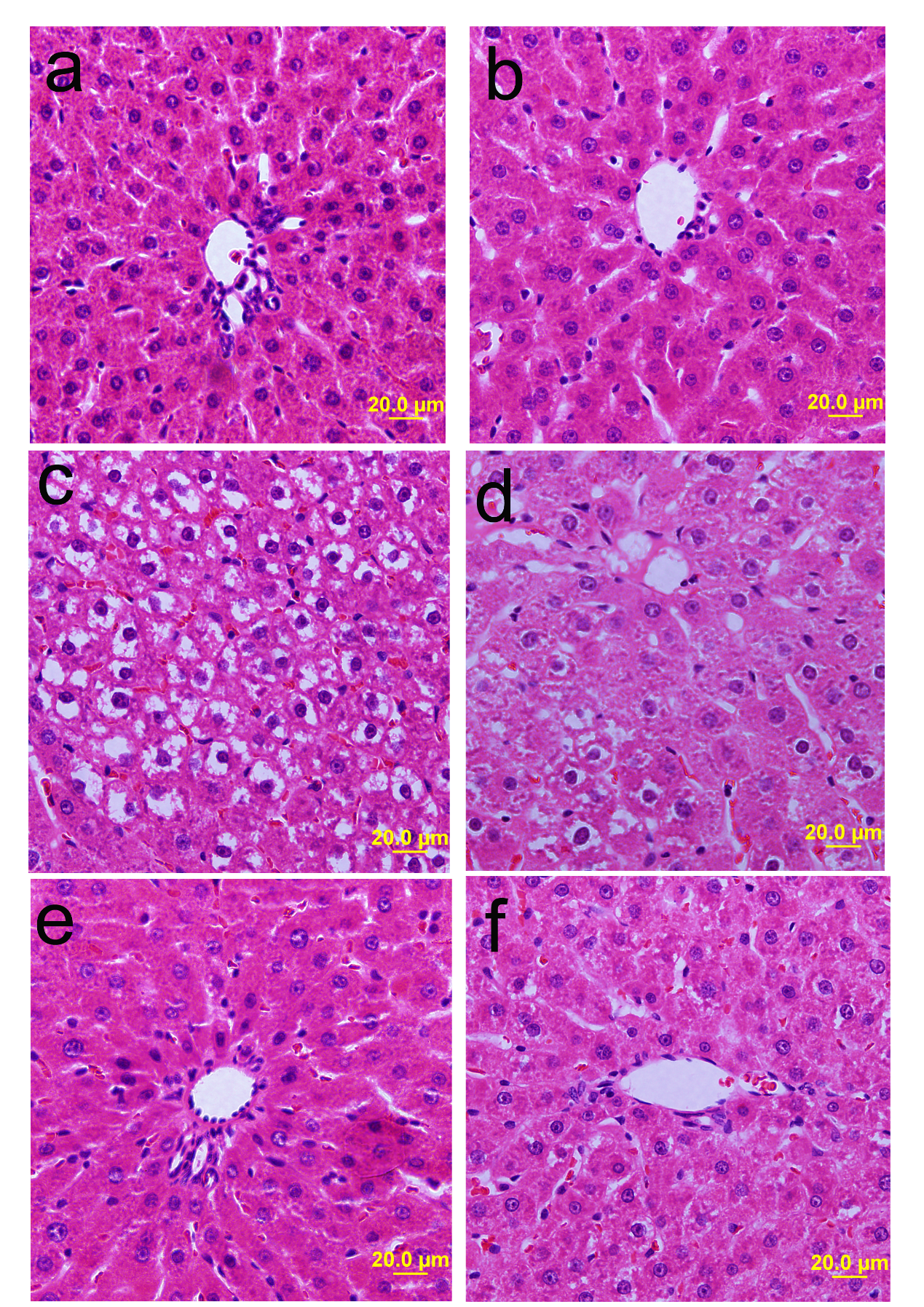
**

**Supplementary Figure 1**. Representative Images of hematoxylin and eosin-stained sections in the livers of all groups studied (Scale bar = 20 µm) a, control, b, treated with high dose of GSE, c, HCC-induced animals, d-f, protected groups treated with low, medium and high dose of GSE.

***Supplementary Figure 1***


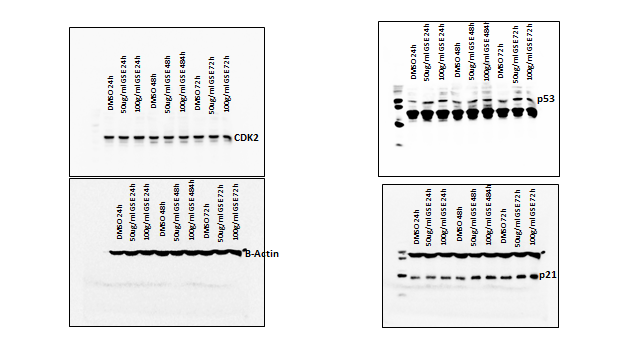


**Supplementary Figure 2. The original blots of figure 6C. *Supplementary Figure 2***

**
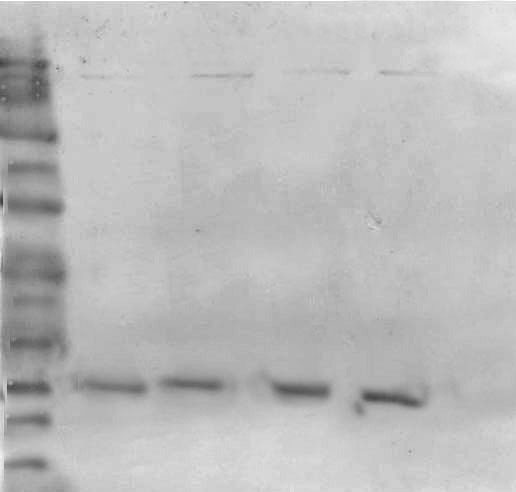
**

0 10 23.9 50 µg/ml

**Bax**

**
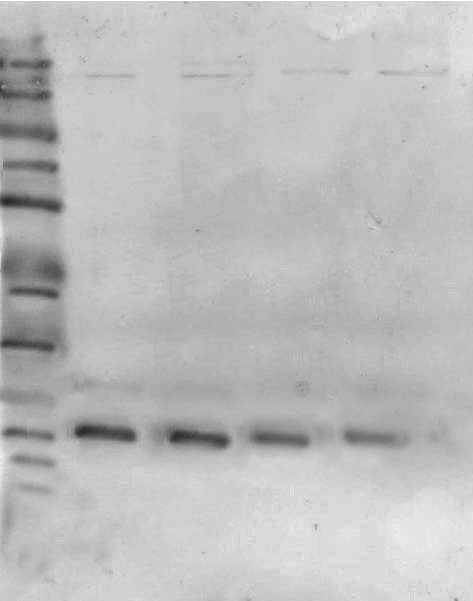
**

0 10 23.9 50 µg/ml

**Bcl-2**

**
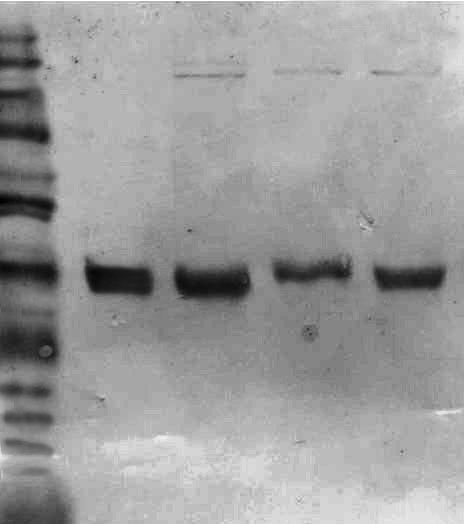
**

0 10 23.9 50 µg/ml

**NF-kB**

**
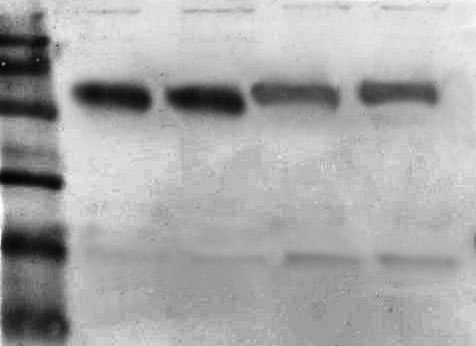
**

**Pro-casp-3**

**Cleaved casp-3**

0 10 23.9 50 µg/ml

**
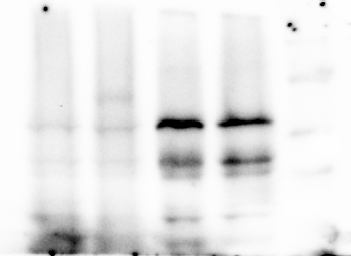
**

0 10 23.9 50 µg/ml

**Cleaved PARP**

***Supplementary Figure 3***

**
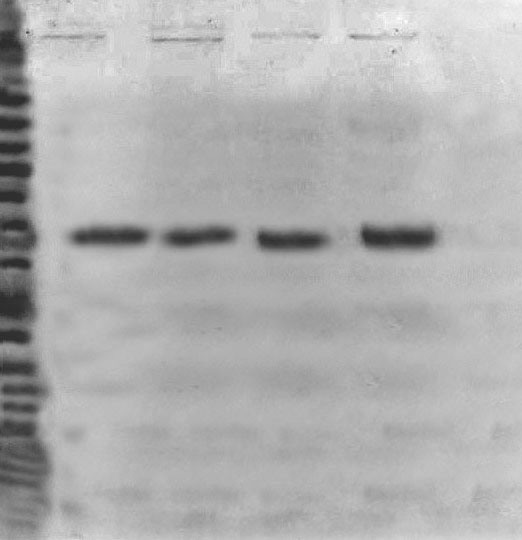
**

0 10 23.9 50 µg/ml

**B-actin**

**Supplementary Figure 3. The original blots of figure 7A.**

**Supplementary Table 1: Effect of GSE on body weight and liver weight of control and DEN-2AAF-**induced liver cancer in male rats.

| **Groups** | **Final Body weight (g)** | | | **Liver weight (g)** | | | **Liver weight/Body weight X 100** | | |
| --- | --- | --- | --- | --- | --- | --- | --- | --- | --- |
| **Control** | 293.3 | ± | 3.80 | 8.73 | ± | 0.18 | 3.10 | ± | 0.20 |
| **GSE** | 290.3 | ± | 2.10 | 8.66 | ± | 0.15 | 3.10 | ± | 0.18 |
| **DEN-2AAF** | 188.3 | ± | 4.94* | 11.01 | ± | 0.14* | 5.82 | ± | 0.12* |
| **DEN-2AAF+GSE LD** | 259.8 | ± | 4.4 *# | 10.9 | ± | 0.14* | 4.17 | ± | 0.06*# |
| **DEN-2AAF+GSE MD** | 287.7 | ± | 1.55# | 10.56 | ± | 0.20* | 3.68 | ± | 0.05*# |
| **DEN-2AAF+GSE HD** | 276.0 | ± | 6.11*# | 10.75 | ± | 0.18* | 3.89 | ± | 0.04*# |

Values are presented as mean ± SME (n = 6). Significance was determined by one-way analysis of variance followed by Dunnett's t-test: * P<0.05 vs. control group,

# P<0.05 vs. DEN-2AAF group

***Supplementary Table 1***
